# Self-Determination Theory in the Social Acceptance of Sustainable Energies and Technologies: A Comprehensive Review

**DOI:** 10.3390/bs16081343

**Published:** 2026-08-04

**Authors:** Mei Xie, Zhuofan Yang, Yiyan Li, Heng Liu, Alessandro Milani, Marino Bonaiuto

**Affiliations:** 1 Department of Psychology of Developmental and Socialization Processes, Sapienza University of Rome, 00185 Rome, Italy; 2 Institute of Applied Psychology, Psychological Research and Counseling Center, Southwest Jiaotong University, Chengdu 610031, China; 3 Inter-University Research Centre in Environmental Psychology (CIRPA), Sapienza University of Rome, 00185 Rome, Italy

**Keywords:** self-determination theory, sustainable energy technologies, technology acceptance, autonomous motivation, social acceptance, renewable energy, psychological needs, pro-environmental behaviour

## Abstract

The transition toward sustainable energy systems is not only a technological and economic challenge but also a social one, with public acceptance playing a decisive role in the success of sustainability transitions. While traditional technology acceptance models primarily emphasize cognitive and utilitarian determinants, increasing attention has been directed toward underlying motivational processes. Self-Determination Theory (SDT) provides a valuable framework for understanding how autonomous and controlled motivation shape attitudes and behaviours toward sustainable energy technologies. This systematic review synthesizes empirical and theoretical research on the application of SDT in the context of social acceptance within this domain. Following PRISMA guidelines, studies published between January 2000 and April 2026 were identified through searches of Web of Science, Scopus, and Google Scholar, with 34 eligible studies included in the thematic analysis. The findings from many of the reviewed studies suggest that autonomous motivation, supported by the satisfaction of autonomy, competence, and relatedness needs, is associated with greater technology acceptance, trust, engagement, and long-term behavioural support. In contrast, controlled motivation is more often associated with resistance, weak internalisation, or short-term compliance. The review further highlights the important moderating role of contextual factors, including policy design, communication strategies, trust, and socio-cultural environments. By integrating SDT into sustainable technology acceptance research, this review advances motivation-based perspectives on sustainability transitions and provides practical implications for policymakers, technology developers, and communication practitioners seeking to foster enduring public engagement with sustainable innovations.

## 1. Introduction

### 1.1. Background

The global transition toward sustainable energy systems has become a defining challenge of the twenty-first century, driven by the urgent need to mitigate climate change, reduce greenhouse gas emissions, and ensure long-term environmental sustainability. According to the Intergovernmental Panel on Climate Change (IPCC, 2023), achieving net-zero emissions requires not only technological innovation but also large-scale societal transformation. In this context, sustainable energy technologies and systems—including renewable energy infrastructures, solar photovoltaics, wind power, smart grids, decentralized storage systems, electric vehicles, and digital energy management technologies—have experienced rapid development and widespread deployment over recent decades (IEA, 2022; Sovacool, 2014). These technologies are increasingly positioned as key solutions for decarbonization, energy security, and sustainable development goals.

However, despite their technical maturity and growing economic viability, the successful implementation of sustainable energy technologies is far from guaranteed. Increasing evidence suggests that technological feasibility alone is insufficient to ensure adoption, diffusion, and long-term public support. Instead, the concept of social acceptance has emerged as a crucial factor shaping the trajectory of energy transitions. As highlighted by Wüstenhagen et al. (2007), social acceptance operates at multiple levels—including socio-political acceptance, community acceptance, and market acceptance—and plays a decisive role in determining whether renewable energy projects succeed or fail. Subsequent studies have further emphasized that public attitudes, trust, and perceived fairness significantly influence acceptance outcomes (Perlaviciute & Steg, 2014; Sovacool et al., 2019).

Empirical examples across different countries further illustrate this point. For instance, large-scale wind farm projects have frequently encountered local resistance due to concerns over landscape disruption, noise pollution, procedural injustice, and insufficient community participation. Similarly, the rollout of smart meters, AI-enabled energy systems, and other digital energy technologies has raised concerns related to privacy, algorithmic transparency, data security, and institutional trust (Malann et al., 2026; Simon & Schweitzer, 2023; Wunderlich et al., 2019). These cases demonstrate that public acceptance may not be solely a rational evaluation of cost–benefit trade-offs. Rather, it is shaped by a complex interplay of social-psychological, contextual, and technological factors (see Bonaiuto et al., 2023, 2024; Dessi et al., 2022; Milani et al., 2024).

As argued by Devine-Wright (2009), opposition to energy projects is often rooted in place attachment, identity concerns, and perceived procedural injustice, rather than purely economic considerations. Similarly, recent research suggests that sustainable transitions increasingly depend not only on technology adoption but also on broader forms of energy citizenship, community participation, and public engagement in energy governance processes (Awopone et al., 2025; Vorobeva et al., 2026). Together, these findings indicate that social acceptance is shaped by complex psychological and social processes that extend beyond purely economic or technological considerations.

This means that the successful diffusion of sustainable energy technologies depends not only on technological feasibility but also on a comprehensive understanding of the psychological and social factors shaping public acceptance. The following section therefore reviews the theoretical perspectives that have been applied to explain these processes.

### 1.2. From Technology Acceptance to Motivation

Traditional models of technology adoption, such as the Technology Acceptance Model (TAM) and the Unified Theory of Acceptance and Use of Technology (UTAUT), have provided valuable insights into how individuals evaluate and adopt new technologies (Davis, 1989; Venkatesh et al., 2003). These frameworks have demonstrated strong predictive power for adoption intentions and perceived usefulness across a wide range of technological contexts. However, the explanatory power of these frameworks appears to be constrained in the specific context of sustainable energy transitions because their primary emphasis on cognitive and instrumental evaluations tends to overlook the deeper motivational processes required to sustain long-term engagement (Claudy et al., 2013; Karbo et al., 2024; Olanipekun et al., 2018). Consequently, while these models effectively address whether an individual might adopt a technology, they offer less insight into why individuals become autonomously motivated—or demotivated—to support sustainable innovations over time. This gap has encouraged researchers to explore complementary theoretical perspectives that prioritize factors such as motivation, values, and psychological needs.

In recent years, scholars have increasingly recognized the importance of motivational and value-based factors in shaping pro-environmental behaviour, technology acceptance, and broader forms of energy citizenship. Research in environmental psychology has highlighted that individuals’ support for sustainable technologies is closely linked to their personal values, environmental beliefs, and moral norms (Steg et al., 2015; van der Werff et al., 2013). For example, individuals who prioritize biospheric values are more likely to support renewable energy initiatives, even in the absence of immediate personal benefits. Another theoretical perspective that has recently gained attention in sustainability research is Conservation of Resources (COR) theory (Hobfoll, 1989; Hobfoll et al., 2018). COR theory posits that individuals strive to acquire, maintain, and protect valued resources, such as time, energy, financial assets, knowledge, and social support. Within the context of sustainable energy transitions, perceptions of resource gain or loss may influence individuals’ willingness to adopt new technologies and engage in energy-related behavioural change. For example, technologies that are perceived as reducing costs, increasing efficiency, or enhancing personal and community resilience may be viewed as resource-enhancing, whereas technologies associated with uncertainty, complexity, or potential risks may be perceived as resource-threatening (Perlaviciute & Steg, 2014; Steg et al., 2014). Consequently, several recent studies have integrated motivational and resource-based perspectives to better understand how psychological needs, perceived benefits, and resource considerations jointly shape sustainable technology acceptance and pro-environmental engagement (Ryan & Deci, 2024; Vansteenkiste et al., 2020).

Moreover, identity-related processes have also been identified as critical drivers of behaviour. People often align their attitudes and actions with their self-concept, social identity, and perceived societal roles (Whitmarsh & O’Neill, 2010). In this sense, acceptance of sustainable technologies is not merely a utilitarian decision but also a reflection of how individuals perceive themselves in relation to broader environmental and societal goals (Jabbour Al Maalouf et al., 2024).

At the same time, emerging smart energy systems—including AI-assisted energy management, gamified energy-saving platforms, peer-to-peer energy trading systems, and smart grids—introduce new psychological challenges related to trust, autonomy, control, and user engagement. These developments further highlight the limitations of purely cognitive acceptance models and reinforce the need for theoretical frameworks capable of capturing the motivational foundations of sustainable technology acceptance. To address this gap and provide a more holistic theoretical perspective, Bonaiuto et al. (2023, 2024) proposed the integrated Sustainable Energy Technology Acceptance (i-SETA) model. The i-SETA model extends traditional technology acceptance frameworks by integrating cognitive, affective, and motivational aspects within the social-psychological determinants of sustainable energy technology acceptance into a unified conceptual framework which considers also technological and contextual aspects as key drivers of social acceptance. Specifically, the model assumes that individuals’ acceptance of sustainable energy technologies is jointly influenced by perceived technological and contextual beliefs (Ariccio et al., 2025), as well as self-perceived personal beliefs. Concerning these categories of beliefs, the model takes into account several factors underlying social acceptance, such as cognitive evaluations (e.g., perceived usefulness and ease of use), affective responses (e.g., positive and negative emotions), motivational processes (e.g., autonomous and controlled motivation); but also broader contextual influences such as social norms, trust, and institutional support. Rather than viewing technology acceptance as a purely rational decision-making process, the i-SETA model conceptualises acceptance as a dynamic psychological process shaped by interactions between individuals and their socio-technical environment. Recent empirical studies have provided growing support for this framework across diverse sustainable energy contexts (e.g., Bonaiuto et al., 2024; Milani et al., 2026a, 2026b). Building upon this theoretical foundation, the present review adopts an SDT perspective to synthesise the motivational mechanisms underlying sustainable energy technology acceptance.

### 1.3. Self-Determination Theory as a Motivational Framework

Self-Determination Theory (SDT), developed by Edward L. Deci and Richard M. Ryan, offers a comprehensive and empirically grounded framework for understanding human motivation (Deci & Ryan, 2000; Ryan & Deci, 2024). Building upon these perspectives, SDT focuses on the quality of motivation and the extent to which behaviour is self-endorsed and aligned with an individual’s values, making it particularly relevant for understanding long-term support for sustainable energy technologies.

A central distinction within SDT is between autonomous motivation and controlled motivation. Autonomous motivation refers to behaviours that are undertaken willingly and are congruent with one’s values and interests. It includes intrinsic motivation, where individuals engage in an activity for its inherent enjoyment, and identified regulation, where individuals recognize the personal importance of a behaviour (Mohamad et al., 2024). In contrast, controlled motivation involves behaviours that are driven by external pressures or internal obligations, such as rewards, punishments, social pressure, or feelings of guilt (Deci et al., 2017; Ryan & Deci, 2000). This distinction is particularly important in sustainable energy contexts, where long-term behavioural engagement often depends on the internalisation of environmental values rather than short-term compliance with external incentives.

In addition to this motivational continuum, SDT posits that human well-being and optimal functioning depend on the satisfaction of three basic psychological needs: autonomy, competence, and relatedness. Autonomy refers to the experience of volition and self-direction; competence involves the perception of effectiveness and mastery; and relatedness reflects the need to feel socially connected and valued by others (Ryan & Deci, 2024; Vansteenkiste et al., 2020).

These psychological needs have been extensively studied across different contexts within environmental psychology (see Ariccio et al., 2021; Mosca et al., 2023; Mura et al., 2024) and are becoming increasingly relevant in the context of sustainable energy acceptance. Individuals are more likely to support renewable energy initiatives and smart energy technologies when they feel that they have meaningful choice and control over energy decisions (autonomy), understand how the technologies function (competence), and perceive that their communities or social groups endorse such behaviours (relatedness) (Cooke et al., 2016; De Groot & Steg, 2010; Perlaviciute & Steg, 2014). Recent studies on energy citizenship, smart grids, and decentralized energy systems further suggest that these psychological needs shape not only adoption intentions but also long-term engagement, participation, and behavioural persistence (Awopone et al., 2025; Brizga & Vijaikis, 2024).

Conversely, policies or technological systems that rely heavily on coercion, excessive surveillance, or purely financial incentives may undermine autonomous motivation and lead to resistance, weak internalisation, or superficial compliance (Pelletier et al., 1998; Steg et al., 2015). This issue is particularly relevant in emerging digital energy systems, where users may perceive reduced autonomy or increased vulnerability due to algorithmic decision-making and data collection practices (Simon & Schweitzer, 2023). These findings further illustrate why SDT provides a particularly suitable framework for examining the social acceptance of sustainable energy technologies, as it captures not only whether individuals adopt new technologies but also why they remain engaged over time.

Importantly, SDT provides a nuanced framework for understanding not only whether individuals accept sustainable technologies, but also the quality, durability, and sustainability of that acceptance over time. Accordingly, the present review adopts SDT as its primary theoretical lens to synthesise existing evidence on the motivational mechanisms underlying the social acceptance of sustainable energy technologies and their implications for long-term behavioural change and sustainability transitions (Pelletier & Sharp, 2008; Ryan & Deci, 2024).

### 1.4. Research Gap

Despite the theoretical relevance of SDT to sustainability and technology acceptance, its application to sustainable energy technologies has developed across multiple disciplinary and technological contexts, with relatively limited integration across these diverse research streams. While a growing number of studies have explored SDT in relation to pro-environmental behaviours, such as energy conservation, recycling, and sustainable consumption, research specifically examining sustainable energy technologies—including renewable energy systems, smart grids, decentralized energy systems, AI-enabled energy management, and energy citizenship—remains scattered across different disciplines and research traditions (Pelletier et al., 1998; Steg et al., 2015). Consequently, a comprehensive synthesis of this expanding body of evidence is still lacking.

Although previous review studies have begun to explore the application of SDT in adjacent domains (e.g., Alberts et al., 2026), their primary focus has been on digital behaviour change interventions, health-related behaviours, or general sustainability practices—or even environmental risk coping behaviours (Ariccio et al., 2021)—rather than on the social acceptance of sustainable energy technologies. Moreover, these reviews generally synthesise SDT applications within individual domains without explicitly examining whether common motivational mechanisms operate across different sustainable energy technologies and socio-technical contexts. In contrast, the present review adopts a broader interdisciplinary perspective by integrating evidence across renewable energy systems, smart grids, decentralized energy systems, electric vehicles, AI-enabled energy management, gamified energy platforms, peer-to-peer energy trading, and energy citizenship. Furthermore, rather than simply summarising previous findings, this review develops an SDT-based integrative conceptual framework that synthesises the relationships among autonomous motivation, controlled motivation, basic psychological need satisfaction, contextual moderators, and sustainable technology acceptance outcomes. In doing so, the review extends the application of SDT within sustainable energy research and provides a clearer theoretical foundation for future empirical studies.

Furthermore, there is still limited understanding of how SDT interacts with contextual moderators such as policy design, institutional trust, social norms, technological complexity, and cultural environments. These factors may significantly shape the relationship between motivation and technology acceptance, yet they are rarely examined systematically or comparatively (Perlaviciute & Steg, 2014; Sovacool et al., 2019).

Finally, although recent studies increasingly discuss concepts such as energy citizenship, participatory governance, and long-term public engagement, there remains a lack of integrative reviews explaining how SDT can contribute to understanding these broader dimensions of sustainability transitions beyond simple technology adoption.

Taken together, these limitations highlight the need for a comprehensive systematic review capable of synthesising existing evidence, identifying recurring motivational mechanisms, clarifying contextual influences, integrating findings across diverse sustainable energy technologies and socio-technical contexts, and proposing an SDT-based conceptual framework to advance future research on the social acceptance of sustainable energy technologies.

### 1.5. Aim of the Study

In response to the gaps identified above, this study aims to provide a comprehensive systematic review of the application of SDT in the context of the social acceptance of sustainable energy technologies and systems.

Specifically, the objectives of this review are fourfold. First, it seeks to systematically synthesise existing empirical and theoretical studies that apply SDT to sustainable technology acceptance, energy citizenship, and public engagement in sustainability transitions, thereby providing an integrated overview of the field. Second, it aims to identify the key motivational mechanisms—such as autonomous motivation, controlled motivation, and psychological need satisfaction—that influence individuals’ attitudes and behaviours toward sustainable energy technologies. Third, the review examines the role of contextual moderators, including social, cultural, institutional, and policy-related factors, in shaping these motivational processes. Finally, it aims to outline future research directions and propose a conceptual framework that can guide subsequent interdisciplinary studies on motivation and sustainable technology acceptance.

By addressing these objectives, this review contributes not only to the advancement of SDT within sustainability research but also to the broader understanding of how motivational processes influence long-term public engagement with sustainable energy systems. In addition, the review offers practical implications for policymakers, technology developers, communication practitioners, and community stakeholders seeking to foster enduring public support for sustainability transitions.

## 2. Methodology

### 2.1. Research Design

This study adopts a systematic review approach to synthesise and critically evaluate the existing literature on the application of SDT in the context of social acceptance of sustainable energy technologies. A systematic review is particularly appropriate for this research as it allows for a transparent, replicable, and comprehensive integration of findings across multiple studies, thereby reducing bias and enhancing the reliability of conclusions (Petticrew & Roberts, 2006; Snyder, 2019). Similar review-based approaches have recently been used to synthesise SDT research in related domains, such as behaviour change technologies (Alberts et al., 2026).

This review was conducted and reported in accordance with the PRISMA 2020 statement and the PRISMA Extension for Scoping Reviews (PRISMA-ScR) where applicable (Page et al., 2021; Tricco et al., 2018). The review process followed the PRISMA framework for identifying, screening, assessing eligibility, and reporting included studies. The PRISMA framework ensures methodological rigor by clearly documenting each stage of the review process, including search strategy, inclusion and exclusion criteria, and data extraction procedures. By adhering to these standards, the present study aims to achieve both transparency and reproducibility, which are essential for high-quality academic research.

Furthermore, given the interdisciplinary nature of the topic—spanning environmental psychology, energy policy, and technology acceptance—the systematic review design enables the integration of diverse theoretical perspectives while maintaining conceptual coherence. This approach is particularly valuable for identifying overarching patterns, theoretical gaps, and emerging trends within the literature (Tranfield et al., 2003). The present systematic review was not registered in PROSPERO, Open Science Framework (OSF), INPLASY, or any other review registry. This was because the review was initiated as an independent systematic literature review rather than as a commissioned or prospectively registered evidence synthesis. Nevertheless, the review was conducted and reported in accordance with the PRISMA 2020 guidelines, with predefined eligibility criteria, a transparent search strategy, and a structured screening procedure.

### 2.2. Search Strategy

A systematic search strategy was developed and iteratively refined to identify studies relevant to the objectives of this review. Three major academic databases were selected for this review: Web of Science, Scopus, and Google Scholar. These databases were chosen because of their extensive coverage of high-quality peer-reviewed publications across disciplines, including social sciences, environmental studies, and technology research (Gusenbauer & Haddaway, 2020).

The search strategy combined keywords related to SDT (e.g., “Self-Determination Theory”, “SDT”, and “intrinsic motivation”), sustainable energy and technologies (e.g., “renewable energy”, “energy transition”, and “green technology”, and specific sustainable energy technologies where relevant), and technology acceptance (e.g., “acceptance”, “adoption”, and “intention”). Keywords within each domain were combined using OR, whereas different domains were combined using AND. The search terms were refined during the search process to improve search sensitivity and relevance while maintaining the focus of the review (Booth et al., 2016).

To further complement the database search, backward and forward citation tracking was conducted. This involved examining the reference lists of key articles and identifying studies that cited these articles, thereby helping identify additional relevant studies that might not have been retrieved through the database searches (Wohlin, 2014). Although truncation symbols and wildcard operators were not employed, this complementary approach helped broaden the scope of the search by identifying potentially relevant studies beyond the database searches. During this process, conceptually related terms and expressions (e.g., “clean energy”, “low-carbon innovation”, and “pro-environmental behaviour”) were also considered when screening references and evaluating the eligibility of potentially relevant studies.

Given the interdisciplinary nature of sustainable energy research, we acknowledge that the database search strategy may not have captured all relevant terms or terminology variations. Additionally, owing to the rapidly evolving nature of this research field, some relevant studies using alternative terminology may not have been identified. This limitation has been acknowledged in the Discussion and should be considered when interpreting the findings of the present review.

Regarding the scope of the search, the literature search covered studies published between January 2000 and April 2026. Only studies that were available online or indexed in the selected databases before this date were considered for inclusion. This period was chosen because it captures the rapid development of both SDT applications and sustainable energy technologies over the past two decades. It also reflects the increasing scholarly attention to the psychological dimensions of environmental behaviour in recent years (Steg et al., 2015).

### 2.3. Inclusion Criteria

To ensure the relevance and quality of the selected studies, a set of inclusion criteria was established prior to the screening process. First, only peer-reviewed journal articles were included in the review. This criterion ensures that the studies have undergone rigorous academic evaluation and meet established standards of research quality (Petticrew & Roberts, 2006).

Second, only studies published in English were considered. While this may limit the inclusion of research conducted in other languages, it ensures consistency in analysis and interpretation and reduces the risk of misinterpretation due to translation issues (Booth et al., 2016). Third, the review included both empirical and theoretical studies, provided that they explicitly applied or discussed SDT. Including theoretical studies was considered important because the application of SDT to sustainable energy technologies remains an emerging and interdisciplinary field. In addition to providing empirical evidence, theoretical contributions help clarify key concepts, propose explanatory mechanisms, identify research gaps, and develop integrative frameworks that can guide future empirical research.

Finally, studies were required to focus on sustainable energy or environmental technologies, such as renewable energy systems, smart energy solutions, or low-carbon innovations. This ensures that the review remains closely aligned with its central research objective of examining SDT within the context of sustainability transitions (Perlaviciute & Steg, 2014).

### 2.4. Exclusion Criteria

In parallel with the inclusion criteria, a set of exclusion criteria was established to remove studies that were not directly aligned with the research objectives.

First, studies that did not explicitly engage with SDT or its core constructs (i.e., autonomy, competence, and relatedness) were excluded. Importantly, studies that applied SDT only at a macro or organisational level—such as those focusing on firm strategies, supply chain management, or institutional performance—without examining individual-level psychological processes were also excluded. For example, research that frames SDT in terms of organisational value internalisation or strategic outcomes, but does not investigate individuals’ motivation, perceptions, or behaviours, was considered outside the scope. This criterion ensures that the review remains focused on individual motivational mechanisms rather than abstract or collective interpretations of the theory (Petticrew & Roberts, 2006).

Second, purely technical or engineering studies were excluded, particularly those focusing exclusively on the design, performance, or efficiency of technologies without incorporating human behaviour, motivation, or psychological mechanisms. While such studies are valuable for technological advancement, they fall outside the scope of the present review, which centres on social acceptance and motivation (Tranfield et al., 2003).

Third, non-peer-reviewed sources—including conference abstracts, reports, dissertations, and other forms of grey literature—as well as review articles (e.g., systematic reviews, scoping reviews, narrative reviews, and meta-analyses) were excluded. Review articles were excluded because this review aimed to synthesise evidence from primary empirical studies and original theoretical studies rather than secondary evidence syntheses (Booth et al., 2016; Snyder, 2019).

Together, these exclusion criteria help refine the dataset and ensure that the final sample consists of studies that are both theoretically relevant and methodologically robust, with a clear focus on individual-level motivational dynamics.

### 2.5. Screening Process

The screening process was conducted in a systematic and stepwise manner to identify the final set of studies included in the review. The process consisted of three main stages, following established guidelines for systematic reviews (Page et al., 2021).

In the first stage, titles and abstracts of all retrieved records were screened to eliminate clearly irrelevant studies. At this stage, studies that did not mention SDT or were unrelated to sustainable technologies were removed. This initial screening helped to significantly reduce the number of articles while retaining potentially relevant studies (Petticrew & Roberts, 2006).

In the second stage, the full texts of the remaining articles were reviewed in detail. This step involved assessing each study against the predefined inclusion and exclusion criteria. Particular attention was paid to the theoretical framework, research context, and methodological approach to ensure alignment with the objectives of the review (Tranfield et al., 2003).

In the final stage, a comprehensive eligibility check was conducted to confirm the inclusion of each study. Any ambiguities or borderline cases were carefully evaluated to ensure consistency in decision-making. This rigorous process helps to minimise selection bias and enhances the credibility of the review (Page et al., 2021).

**Figure 1:**
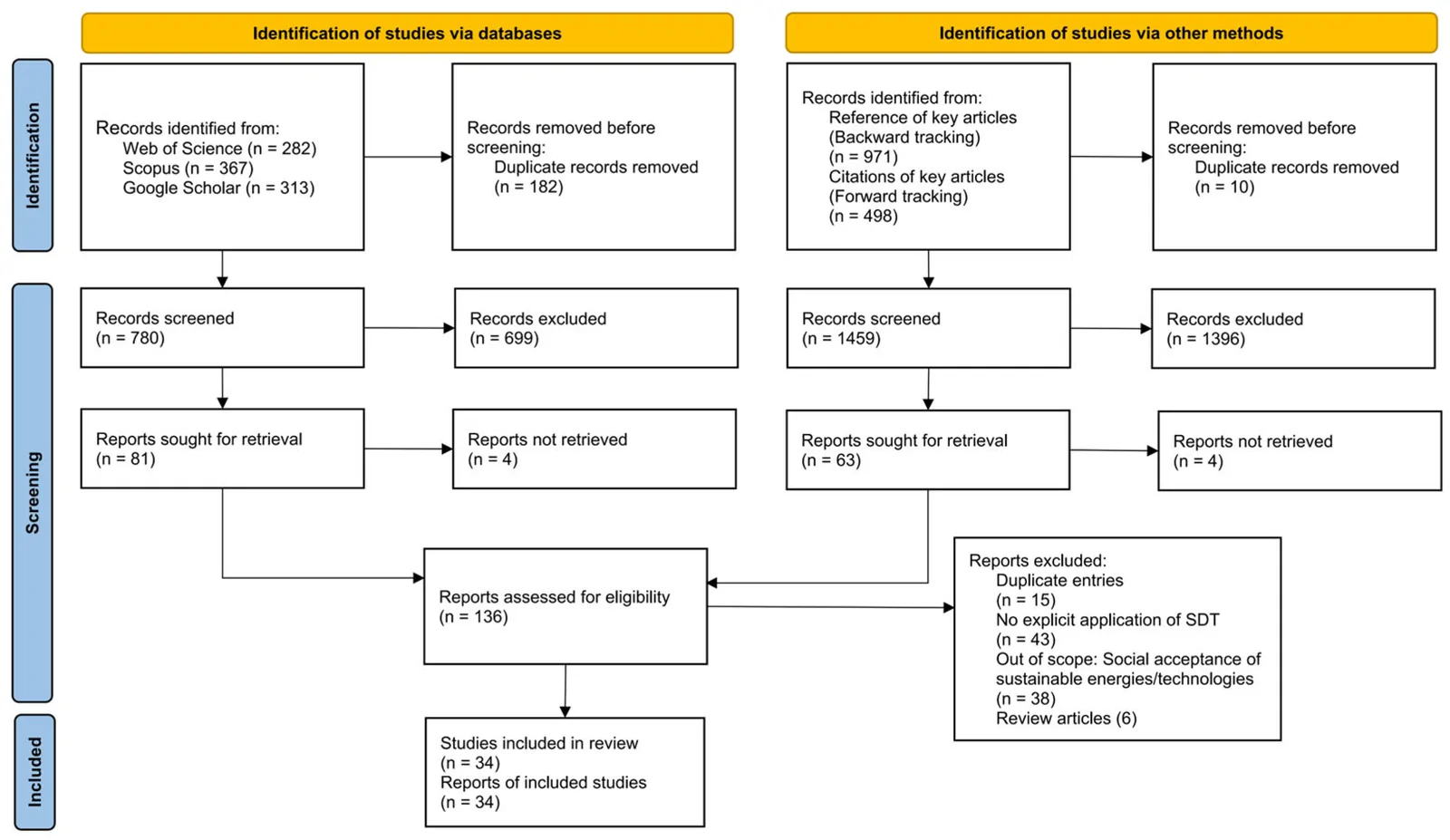
PRISMA flow diagram.

A PRISMA flow diagram visually illustrates the screening process, including the number of records identified, screened, excluded, and included at each stage (Figure 1).

### 2.6. Data Extraction and Analysis

Following the identification of the final set of included studies, a structured data extraction procedure was conducted. Key information was systematically recorded for each study—including the research context, sample characteristics, methodological design, specific approaches, SDT constructs examined, and main findings (Booth et al., 2016). To evaluate the methodological quality of the included evidence, all empirical studies were assessed using the Mixed Methods Appraisal Tool (MMAT, version 2018; Hong et al., 2018). The appraisal was independently conducted by two reviewers, and disagreements were resolved through discussion and consensus. These procedures enhance methodological transparency and support a systematic synthesis of evidence across diverse study designs.

The extracted data were then analysed using a thematic analysis approach. Thematic analysis is particularly suitable for this study because it allows for the identification of recurring patterns and themes across diverse studies. Through an iterative process of coding and categorisation, key themes related to motivation, psychological needs, and social acceptance were identified and synthesised (Braun & Clarke, 2006; Clarke & Braun, 2017).

In addition, the analysis paid attention to variations across contexts, such as differences in cultural settings, policy environments, and types of technologies. This allows for a more nuanced understanding of how SDT operates under different conditions.

Overall, this methodological approach provides a foundation for synthesising the literature and generating meaningful insights into the role of SDT in the social acceptance of sustainable energy technologies.

## 3. Results

### 3.1. Overview of Included Studies

**Figure 2:**
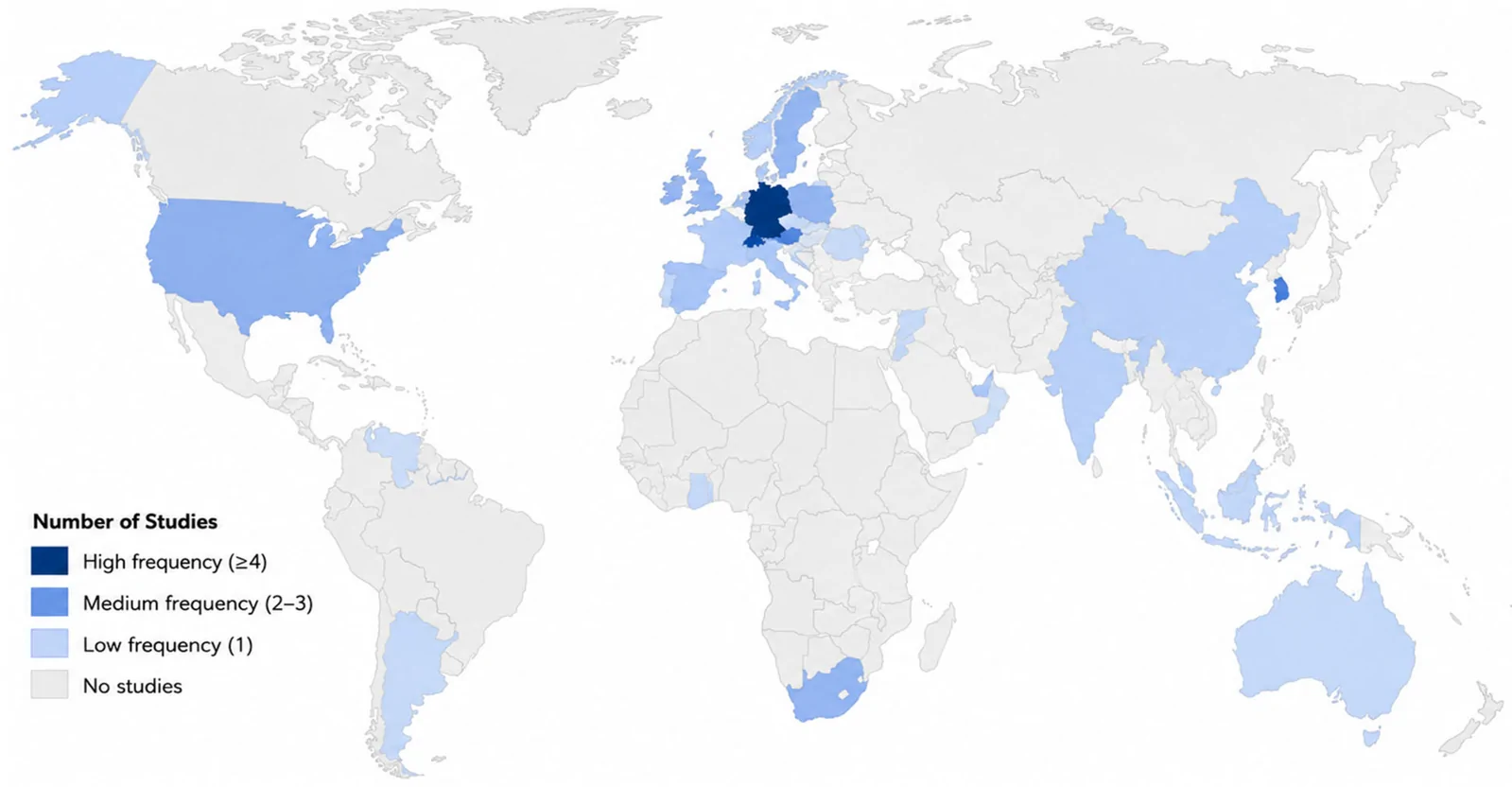
Frequency of included studies by country.

The final sample comprised 34 peer-reviewed studies examining the role of SDT in the social acceptance, adoption, and engagement of sustainable energy technologies and systems across diverse geographical and technological contexts. A detailed overview of all included studies, including their methodological characteristics, technological contexts, and main findings, is provided in Appendix A. As illustrated in Figure 2, the included studies demonstrate broad geographical coverage, spanning Europe (e.g., the Netherlands, Germany, Belgium, Sweden, Switzerland, France, Austria, Norway, Latvia, Portugal, Italy, and the United Kingdom), Asia (e.g., South Korea, China, India, Jordan, Qatar, Malaysia, Indonesia, and Bangladesh), Africa (e.g., Ghana and South Africa), North America (e.g., the United States), as well as several multi-country and cross-regional investigations. Cross-national studies include comparisons across European countries, Europe and Latin America, and international samples, reflecting increasing interest in understanding SDT-based motivational processes across diverse sociotechnical contexts. Despite this broad international representation, European settings remain particularly prominent, likely reflecting the region’s longstanding policy commitment to the energy transition, widespread deployment of renewable energy technologies, mature smart-grid infrastructures, and active promotion of decentralized energy systems.

Methodologically, the reviewed literature is highly diverse. Despite this diversity, the methodological quality appraisal indicated that most empirical studies demonstrated moderate to high methodological quality based on the MMAT assessment. A large proportion of studies employed quantitative survey designs combined with advanced statistical modelling approaches such as structural equation modelling (SEM), partial least squares SEM (PLS-SEM), confirmatory factor analysis (CFA), hierarchical regression, and necessary condition analysis (NCA) (e.g., Vorobeva et al., 2026; S. Wang et al., 2024; Wunderlich et al., 2013; H. Zhao et al., 2024). Several studies adopted mixed-methods designs that combined surveys with interviews, Delphi panels, or stakeholder workshops (e.g., Aguayo-Mendoza et al., 2024; Bögel et al., 2021; Ecker et al., 2017). In addition, the literature includes qualitative interview studies exploring motivational experiences and barriers in everyday energy practices (e.g., Chikandiwa & Mutambara, 2023; Murtagh et al., 2014; Thunshirn et al., 2025), experimental studies examining motivational crowding and remuneration effects (Stauch & Gamma, 2020), conceptual and theoretical analyses (Joachain & Klopfert, 2014; Mohamad et al., 2024; Olanipekun et al., 2018), as well as systematic and narrative reviews focused on gamification, AI-enabled energy systems, and behavioural engagement (Jufri et al., 2026; Malann et al., 2026). The dataset also contains several interdisciplinary technical and optimization-oriented studies integrating SDT with artificial intelligence, gamification, differentiable robust optimization, and deep reinforcement learning frameworks for smart energy systems and peer-to-peer trading environments (Y. Wang et al., 2025; Zhang et al., 2025; A. P. Zhao et al., 2025).

The reviewed studies also vary considerably in scale and participant composition. Sample sizes range from small exploratory qualitative studies involving 7–26 participants (e.g., Flostrand et al., 2019; Thunshirn et al., 2025) to large-scale surveys involving more than 1000 respondents (e.g., Peng & Klöckner, 2026; Simon & Schweitzer, 2023; S. Wang et al., 2024). Participants include homeowners, rural residents, energy consumers, university academics, EV users, policy stakeholders, and prosumers, reflecting the broad societal relevance of sustainable energy transitions.

**Figure 3:**
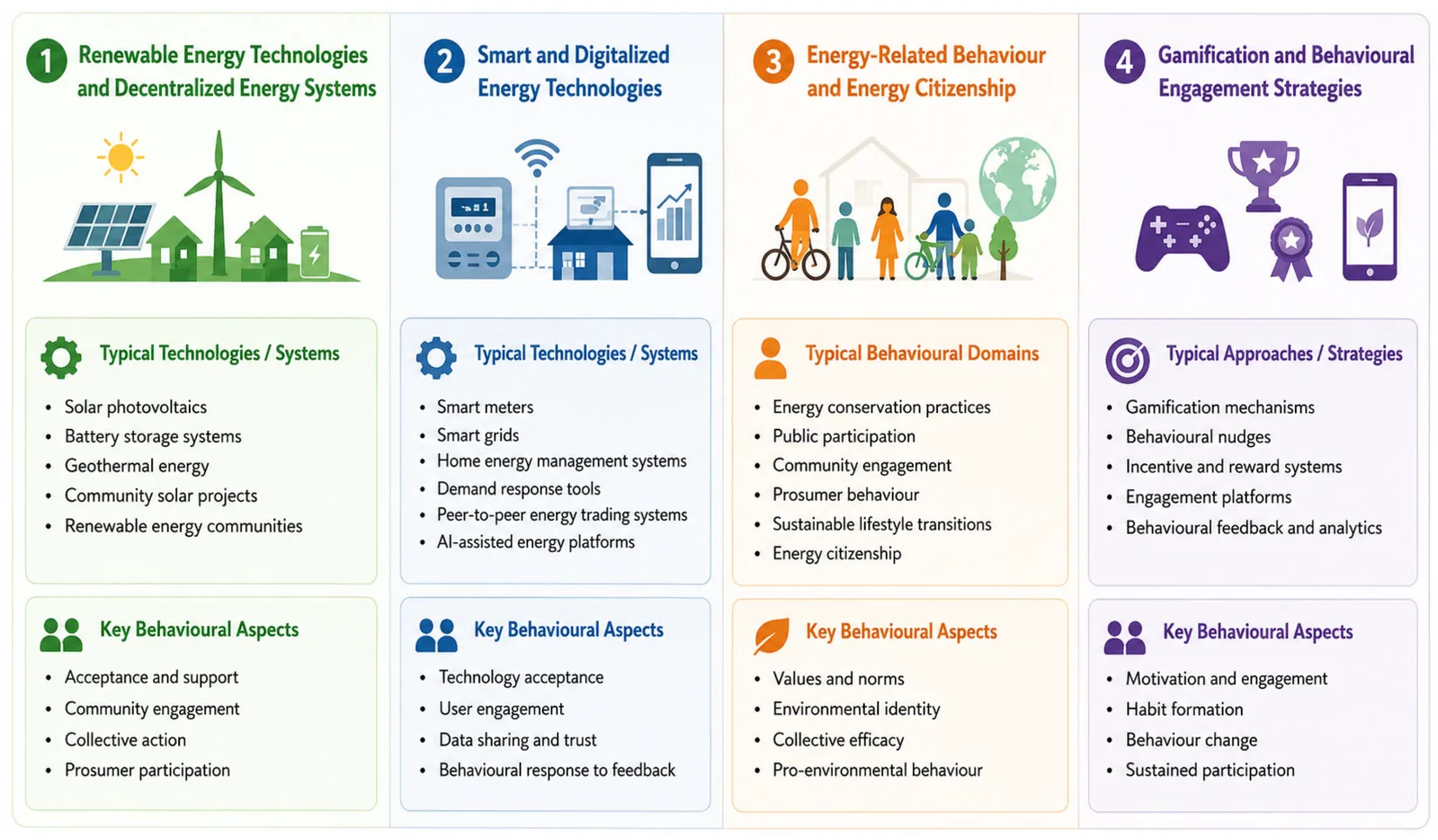
Thematic clusters of sustainable energy technologies and behavioral domains (based on included studies).

In terms of thematic focus, the literature spans multiple technological and behavioural domains (Figure 3). First, a substantial body of research focuses on renewable energy technologies and decentralized energy systems, including solar photovoltaics, battery storage systems, geothermal energy, community solar projects, and renewable energy communities (Ecker et al., 2017, 2018; Legault et al., 2024; Tirotto & Pahl, 2023). Second, a rapidly growing stream of studies investigates smart and digitalized energy technologies, such as smart meters, smart grids, home energy management systems, demand response tools, peer-to-peer energy trading systems, and AI-assisted energy platforms (Malann et al., 2026; Simon & Schweitzer, 2023; Wunderlich et al., 2013, 2019; Zhang et al., 2025). Third, several studies extend beyond technology adoption to examine broader forms of energy-related behaviour and energy citizenship, including conservation practices, public participation, community engagement, prosumer behaviour, and sustainable lifestyle transitions (Awopone et al., 2025; Brizga & Vijaikis, 2024; Vorobeva et al., 2026). Finally, a growing number of recent studies explore gamification and behavioural engagement strategies within smart energy systems, suggesting an increasing convergence between sustainability transitions, behavioural science, and digital innovation (Jufri et al., 2026).

Across these diverse domains, the majority of empirical studies suggest that psychological and motivational factors grounded in SDT are important contributors to individuals’ acceptance, adoption intentions, behavioural persistence, and long-term engagement with sustainable energy technologies and systems. More specifically, autonomous forms of motivation, psychological need satisfaction, and value internalisation repeatedly emerge as stronger predictors of sustained engagement than purely external incentives or instrumental considerations. This suggests that sustainable energy transitions are not solely technological or economic processes, but also deeply motivational and psychological transformations requiring active public participation and internalised commitment. Although the reviewed studies consistently support the importance of autonomous motivation and psychological need satisfaction, the relative salience of these mechanisms differs across technological and geographical contexts. As summarized in Table 1, these mechanisms vary across four primary energy application domains, reflecting nuanced shifts in psychological needs and motivational pathways. In addition, studies conducted in European countries more frequently emphasise autonomy, participatory governance, and energy citizenship, whereas studies from developing economies place greater emphasis on financial affordability, infrastructure, and policy support as important conditions shaping motivational processes. Likewise, AI-supported and smart energy systems increasingly highlight trust, transparency, and user control, while studies of conventional renewable energy technologies focus more strongly on environmental values and behavioural internalisation.

### 3.2. Autonomous Motivation and Acceptance

A central finding across most studies is that autonomous motivation is frequently identified as a significant predictor of acceptance and adoption of sustainable technologies. Autonomous motivation—encompassing intrinsic motivation and internalised extrinsic motivation—reflects behaviours aligned with personal values and self-endorsed goals.

Evidence from multiple empirical studies shows that individuals with higher levels of autonomous motivation are more likely to adopt and support sustainable energy technologies. For instance, Wunderlich et al. (2013) found that internal motivation strongly predicts adoption intentions for smart energy services, while external motivation plays a weaker role. Similarly, Legault et al. (2024) demonstrate that pro-environmental autonomous motivation robustly predicts residential solar adoption across demographic groups.

Autonomous motivation is closely linked to value congruence and environmental identity. Individuals who perceive sustainable technologies as aligned with their environmental values are more willing to adopt them, even when costs are involved. Brizga and Vijaikis (2024) further show that intrinsic personal and social motivations significantly predict energy citizenship, while extrinsic motivations are weak or even negative predictors.

Importantly, autonomous motivation also contributes to sustained engagement over time. Webb et al. (2025) identify consumer segments and show that individuals with high autonomous motivation exhibit the strongest habitual energy-saving behaviours, highlighting the long-term effectiveness of self-determined motivation.

In addition, qualitative evidence supports these findings. Thunshirn et al. (2025) report that motivations such as autonomy, personal control, and pro-environmental values are key drivers of active smart meter usage, indicating that self-determined motivation operates consistently across both behavioural and technological contexts.

Taken together, these findings suggest that autonomous motivation tends to be a reliable predictor across the various categories of sustainable energy technologies analyzed in this review. Nevertheless, its expression varies across contexts. In conventional renewable energy applications, autonomous motivation is primarily associated with environmental values and personal responsibility, whereas in AI-supported and smart energy systems it is more closely related to perceived autonomy, transparency, and user empowerment.

### 3.3. Role of Basic Psychological Needs

The majority of the empirical studies reviewed provide evidence supporting the importance of the three basic psychological needs proposed by SDT—autonomy, competence, and relatedness—in shaping individuals’ acceptance, adoption, and sustained engagement with sustainable energy technologies and systems. Although the relative importance of each need varies across technological and sociocultural contexts, the findings collectively suggest that sustainable energy initiatives appear more likely to achieve public engagement when these psychological needs are supported rather than constrained.

#### 3.3.1. Autonomy

The need for autonomy emerges as a prominent psychological driver in most of the examined contexts of sustainable energy acceptance. Across multiple studies, individuals show stronger support for technologies and energy systems that enhance personal control, self-sufficiency, and freedom of choice in energy production and consumption.

Research on decentralized energy systems frequently highlights the importance of autonomy in shaping individuals’ acceptance of sustainable energy technologies. Ecker et al. (2017) found that individuals prefer household-level decentralized energy systems because they provide greater feelings of independence, autonomy, control, and energy security. These autonomy-related perceptions significantly increase willingness to invest in photovoltaic systems and energy storage technologies. Similar findings were reported by Ecker et al. (2018), who demonstrated that perceived energy autarky strongly increases willingness to pay for private energy storage systems, even more than economic considerations alone.

The importance of autonomy also extends to smart and digitalized energy systems. Bögel et al. (2021) found that citizens value autonomy within decentralized energy infrastructures but simultaneously prefer low-effort and automated solutions that reduce cognitive burden. This suggests that users seek a balance between maintaining meaningful control and minimizing excessive technological complexity. Likewise, Thunshirn et al. (2025) identified self-determination, personal control, and autonomy as key motivations for active smart meter use, particularly in relation to energy monitoring and participation in energy communities.

Several studies further demonstrate that autonomy is central to emerging forms of energy citizenship and participatory energy governance. Tirotto and Pahl (2023) showed that group-level autonomy significantly enhances the social acceptance of geothermal energy projects through procedural justice and collective self-determined motivation. Similarly, Vorobeva et al. (2026) found that intrinsic motivation and identified regulation positively influence participation in energy communities and demand response systems. These findings suggest that autonomy operates not only at the individual level but also collectively through participation, procedural fairness, and community empowerment.

Autonomy is also increasingly embedded in recent AI-supported and gamified energy systems. Technical and conceptual studies integrating SDT with peer-to-peer energy trading, gamification, and AI-driven smart grids consistently position autonomy as a central mechanism for user engagement and behavioural participation (Y. Wang et al., 2025; Zhang et al., 2025; A. P. Zhao et al., 2025). In these contexts, autonomy involves both behavioural freedom and users’ perceived agency within increasingly automated digital systems.

#### 3.3.2. Competence

The need for competence refers to individuals’ perceived ability to understand, manage, and effectively interact with sustainable energy technologies. Across the reviewed literature, competence is identified in many of the studies as a critical factor influencing acceptance, internalisation, and continued technology use.

Several studies indicate that technological knowledge and understanding strengthen autonomous motivation and long-term engagement. Koo and Chung (2014) found that eco-technological knowledge significantly enhances intrinsic and autonomous extrinsic motivation, which subsequently promotes continued use of smart green IT and energy-saving technologies. Similarly, Aguayo-Mendoza et al. (2024) identified competence as one of the most important drivers of household investment decisions in energy-transition technologies, often exceeding the importance of purely financial considerations.

In the context of renewable energy adoption, S. Wang et al. (2024) demonstrated that residential photovoltaic installation increases individuals’ perceptual competence and ownership consciousness, which in turn strengthens willingness to participate in coal-to-electricity transitions. H. Zhao et al. (2024) likewise found that competence positively influences innovative consumption behaviours and electric vehicle purchase intentions among Chinese consumers. These findings suggest that individuals are more likely to engage with sustainable technologies when they feel capable of understanding and effectively using them.

Conversely, competence frustration and technological uncertainty often undermine engagement. Simon and Schweitzer (2023) found that perceived data vulnerability associated with smart meters reduces users’ sense of competence and psychological empowerment, thereby weakening the internalisation of energy-transition goals. Likewise, Thunshirn et al. (2025) identified information deficits, cognitive overload, and perceived uselessness as major barriers preventing active smart meter engagement. Similar barriers were reported by Chikandiwa and Mutambara (2023), who found that homeowners often perceive renewable energy technologies as overly complex and difficult to navigate, especially in the absence of adequate support networks.

Recent studies on AI-powered and gamified energy systems further reinforce the importance of competence. Gamification frameworks and AI-assisted platforms often aim to enhance users’ perceived competence through feedback systems, performance tracking, rewards, and interactive learning environments (Malann et al., 2026). However, these studies also caution that excessive technological complexity, algorithmic opacity, and poor system transparency may undermine users’ sense of mastery and reduce long-term engagement.

#### 3.3.3. Relatedness

The need for relatedness refers to the desire to feel socially connected, valued, and embedded within meaningful social relationships and communities. Across the reviewed studies, relatedness emerges as a important mechanism linking social norms, collective identity, trust, and community participation to sustainable energy acceptance.

Community-oriented and participatory approaches promote engagement by fostering social belonging and collective involvement. Bögel et al. (2021) found that social trust and local sharing norms significantly influence preferences for shared versus individual energy storage systems. In Sweden, participants were more willing to adopt shared energy infrastructures when supported by strong social trust and collaborative norms, whereas participants in Portugal preferred more individualized solutions.

The importance of relatedness is especially visible within the growing literature on energy citizenship and community participation. Brizga and Vijaikis (2024) demonstrated that social intrinsic motivations significantly predict energy citizenship and future willingness to participate in sustainable energy transitions. Similarly, Awopone et al. (2025) found that community engagement in renewable energy initiatives is strongly influenced by motivation, perceived collective benefits, and social participation. These findings indicate that sustainable transitions are facilitated when individuals perceive themselves as part of broader collective efforts and socially meaningful environmental movements.

Relatedness also operates through procedural justice and social identity processes. Tirotto and Pahl (2023) showed that collective self-determined motivation and procedural justice climates significantly increase social acceptance of geothermal energy projects, whereas social identity violations undermine acceptance. Similarly, Vorobeva et al. (2026) found that behavioural intention and participation in energy communities are strengthened when users experience trust and social connectedness within demand response systems.

In addition, recent studies suggest that relatedness is increasingly incorporated into gamified and AI-supported energy systems. Gamification strategies such as leaderboards, collaborative challenges, social rewards, and community clustering are frequently designed to foster social engagement and collective participation in sustainable behaviours (Jufri et al., 2026; Y. Wang et al., 2025; A. P. Zhao et al., 2025). These approaches indicate that social connectedness remains highly relevant even within technologically advanced and digitally mediated sustainability systems.

Taken together, the reviewed literature provides substantial support for the SDT proposition that autonomy, competence, and relatedness are fundamental psychological mechanisms underlying sustainable technology acceptance. However, the relative importance of each psychological need appears to vary across socio-technical contexts. For example, competence is particularly salient in technologically complex systems such as AI-assisted platforms and smart grids, whereas relatedness becomes more prominent in community energy projects and energy citizenship initiatives.

### 3.4. Controlled Motivation and Resistance

In contrast to autonomous motivation, controlled motivation generally demonstrates weaker, inconsistent, or even negative effects on sustainable energy acceptance and long-term engagement. Within SDT, controlled motivation refers to behaviours driven primarily by external rewards, social pressure, regulatory obligations, or avoidance of guilt and punishment. Although controlled motivation may sometimes encourage initial adoption, the reviewed studies consistently suggest that it is less effective in promoting durable behavioural commitment and may even undermine intrinsic engagement under certain conditions.

A recurring finding across several empirical studies is that purely financial incentives often fail to generate meaningful long-term engagement and may occasionally crowd out intrinsic motivation. Stauch and Gamma (2020) demonstrated that environmentally motivated consumers prefer solar-energy remuneration over direct financial compensation in community solar projects. Financial rewards were found to reduce intrinsic motivation among environmentally oriented participants, whereas symbolic and sustainability-oriented incentives generated stronger engagement. Similarly, Joachain and Klopfert (2014) argued that smart-meter systems relying on symbolic rewards and value-oriented incentives are more effective than purely financial mechanisms in strengthening autonomous motivation for energy conservation.

Several studies further indicate that excessive dependence on external regulation may lead to superficial compliance rather than genuine internalisation. Dias et al. (2025) found that external motivation predicts electric vehicle adoption intention in India, but identified and integrated regulation exert substantially stronger effects. Likewise, Sugihara and Hardman (2022) reported that electric vehicle purchases within fleet systems are often driven by external sustainability mandates, regulatory pressures, and organizational requirements rather than personally internalised environmental values. These findings suggest that controlled motivation may facilitate short-term adoption while remaining vulnerable to behavioural instability and low long-term commitment.

The reviewed studies also reveal important contextual variations in the effects of controlled motivation. In developing and economically constrained settings, external incentives may play a more significant role in promoting adoption. For example, Al-Habaibeh et al. (2023) found that financial support, subsidies, and low monthly payment schemes are major drivers of domestic solar energy adoption in Jordan, where economic barriers remain substantial. Similarly, S. Wang et al. (2024) reported that economic incentives exert stronger effects on coal-to-electricity transition willingness than environmental campaigns among Chinese rural residents. These findings suggest that controlled motivation cannot be dismissed entirely, particularly in contexts characterized by financial limitations or infrastructural barriers.

However, several studies also indicate that externally controlled systems may weaken intrinsic engagement over time. Al-Marri et al. (2018) showed that heavily subsidized energy systems in Qatar reduce consumers’ motivation for energy conservation because energy costs become psychologically disconnected from personal responsibility and environmental awareness. Awopone et al. (2025) similarly identified a motivational crowding-out effect, whereby perceived benefits weaken the relationship between intrinsic motivation and community engagement. These findings are highly consistent with SDT’s argument that excessive reliance on external rewards or pressure can undermine autonomous forms of motivation and reduce long-term behavioural persistence.

Controlled motivation also appears in the context of smart and digital energy systems, particularly through surveillance concerns, algorithmic control, and perceived loss of autonomy. Simon and Schweitzer (2023) demonstrated that smart meter–related data vulnerability and psychological disempowerment reduce users’ internalisation of energy-transition goals. Similarly, several studies on AI-powered energy systems caution that excessive automation, opaque algorithms, and externally imposed behavioural optimization may create feelings of reduced autonomy and technological dependence (Malann et al., 2026; Zhang et al., 2025). These concerns suggest that digitally mediated sustainability systems may inadvertently generate resistance when users perceive them as controlling rather than empowering.

Taken together, the reviewed literature suggests that controlled motivation may facilitate initial behavioural compliance or short-term adoption under certain economic or institutional conditions, but it rarely produces stable and enduring engagement on its own. In contrast, sustainable long-term participation appears more likely when external incentives and policies support the internalisation of environmental goals and the satisfaction of autonomy, competence, and relatedness needs rather than relying exclusively on external pressure or economic rewards.

### 3.5. Contextual Moderators

The reviewed studies generally indicate that the relationship between SDT constructs and sustainable energy acceptance is influenced by contextual moderators. Although autonomous motivation, psychological need satisfaction, and value internalisation generally promote engagement with sustainable technologies, their effects are neither universal nor context-independent. Instead, motivational processes are significantly influenced by trust, procedural fairness, socio-demographic characteristics, technological design, policy environments, and broader sociocultural conditions.

#### 3.5.1. Trust, Transparency, and Procedural Fairness

Trust emerges as one of the most frequently examined contextual moderators across the reviewed studies, particularly within smart and digitalized energy systems. Several studies suggest that even highly motivated individuals may resist sustainable technologies when they perceive risks related to privacy, surveillance, fairness, or institutional transparency (Streimikiene et al., 2021).

Simon and Schweitzer (2023) demonstrated that perceived data vulnerability associated with smart meters reduces consumers’ sense of competence and psychological empowerment, thereby weakening the internalisation of energy-transition goals. Importantly, the study further showed that transparent communication and fair data practices by electricity suppliers can partially mitigate these negative effects. Similarly, Wunderlich et al. (2019) found that privacy concerns negatively influence the adoption of smart metering technologies, particularly among older users and individuals with lower trust in digital infrastructures.

Procedural fairness and participatory governance also significantly shape motivational outcomes. Tirotto and Pahl (2023) showed that group-level autonomy enhances the social acceptance of geothermal energy projects through procedural justice climates and collective self-determined motivation. Conversely, perceptions of social identity violation strongly reduce public acceptance. These findings suggest that acceptance depends not only on technological benefits but also on whether decision-making processes are perceived as fair, inclusive, and respectful of local identities.

The importance of trust is further reflected in studies on decentralized and community-based energy systems. Bögel et al. (2021) found that social trust and sharing norms significantly influence preferences for collective versus individual energy storage systems. Similarly, Vorobeva et al. (2026) demonstrated that trust positively predicts behavioural intention and participation in energy communities and demand response systems. These findings collectively suggest that trust may operate as an important social condition enabling the internalisation of sustainability goals and collective engagement in energy transitions.

#### 3.5.2. Socio-Demographic and Cultural Moderators

The reviewed studies further indicate that motivational processes vary substantially across demographic, cultural, and socio-economic groups. In many cases, the effects of autonomous and controlled motivation depend on factors such as age, education, income, political orientation, gender, and regional context.

Legault et al. (2024) demonstrated that autonomous pro-environmental motivation consistently predicted residential solar adoption regardless of demographic characteristics, whereas the predictive value of controlled motivation varied according to age, income, education, and political orientation. Specifically, controlled motivation predicted adoption more strongly among younger and higher-income households but was less predictive among older adults and highly educated individuals. Similarly, Dias et al. (2025) found that external and identified motivation predicted electric vehicle adoption intentions more strongly among men, whereas introjected and integrated motivation were more influential among women, highlighting distinct gender-specific motivational pathways to adoption.

Regional and cultural differences also shape the importance of psychological needs. Aguayo-Mendoza et al. (2024) found that European households prioritize autonomy in energy-transition decisions, whereas Latin American households prioritize competence. Additionally, a higher level of education shifts the focus toward autonomy, while lower educational attainment increases reliance on competence as the primary driver for technology adoption. Bögel et al. (2021) likewise identified contrasting energy preferences between Sweden and Portugal, with Swedish participants favoring shared energy systems and Portuguese participants preferring individual solutions, reflecting broader sociocultural differences in trust, collectivism, and energy governance traditions.

Several studies from developing economies additionally demonstrate that economic and infrastructural conditions moderate motivational processes. Al-Habaibeh et al. (2023) found that financial incentives and affordability remain major determinants of solar adoption in Jordan, despite strong environmental awareness. Similarly, S. Wang et al. (2024) reported that economic incentives exert stronger effects than environmental campaigns in promoting coal-to-electricity transitions among rural Chinese residents. These findings suggest that the relative influence of autonomous versus controlled motivation may vary substantially depending on local socio-economic conditions and resource availability.

#### 3.5.3. Technological Characteristics and System Design

Technological characteristics themselves also interact with psychological and motivational processes. This corresponds to recent empirical results about the association of perceived energy technology features with both acceptability attitudes and acceptance intentions (Bonaiuto et al., 2023, 2024; Milani et al., 2024). Across the reviewed literature, factors such as usability, perceived complexity, automation, transparency, cognitive load, and perceived risk shape individuals’ engagement with sustainable technologies.

Thunshirn et al. (2025) identified several barriers to active smart meter engagement, including cognitive overload, low awareness of system functions, perceived uselessness, and information deficits. Similarly, Chikandiwa and Mutambara (2023) found that perceived technological complexity and lack of support networks reduce homeowners’ willingness to adopt microgeneration technologies. These findings indicate that even individuals with strong pro-environmental motivations may disengage when technologies are perceived as difficult to understand or use.

Research on AI-enabled and gamified systems further highlights the importance of technological design. Y. Wang et al. (2025), Zhang et al. (2025), and Malann et al. (2026) emphasize that gamification features, feedback systems, and user-centred AI design can strengthen engagement by enhancing autonomy, competence, and relatedness. However, these studies also caution that excessive automation, opaque algorithms, digital inequality, and privacy concerns may undermine trust and psychological empowerment. In this sense, technological systems do not merely facilitate sustainable behaviour; they actively shape the motivational conditions under which engagement occurs.

The role of system design is particularly visible in studies examining smart grids and demand response technologies. Sintov and Schultz (2015) argue that flexible control systems and behaviourally informed design strategies are more effective than rigid or highly controlling energy-management systems. Likewise, Joachain and Klopfert (2014) suggest that symbolic and value-oriented reward systems are more effective than purely financial incentives in supporting long-term engagement with smart energy technologies.

#### 3.5.4. Policy and Institutional Contexts

Policy and institutional environments also substantially influence motivational processes related to sustainable technology acceptance. This corresponds to recent empirical results about the association of perceived political and financial contextual features with both acceptability attitudes and acceptance intentions (Bonaiuto et al., 2023, 2024; Milani et al., 2024). Across the reviewed studies, policies that support autonomy, participation, fairness, and psychological need satisfaction tend to produce stronger and more enduring engagement than purely informational or coercive approaches.

Awopone et al. (2025) found that policy awareness alone does not significantly predict community engagement in renewable energy initiatives, suggesting that information provision without motivational support is insufficient to foster meaningful participation. Similarly, Al-Marri et al. (2018) reported that heavily subsidized energy systems reduce consumers’ motivation for conservation because energy consumption becomes disconnected from personal responsibility and environmental awareness.

Other studies indicate that policy design can either facilitate or undermine motivational internalisation. Stauch and Gamma (2020) demonstrated that financial compensation may crowd out intrinsic motivation among environmentally motivated consumers, whereas sustainability-oriented reward structures better support engagement. Olanipekun et al. (2018) similarly argued that sustainability policies should be tailored to different motivational levels and gradually support movement toward more self-determined forms of motivation.

**Figure 4:**
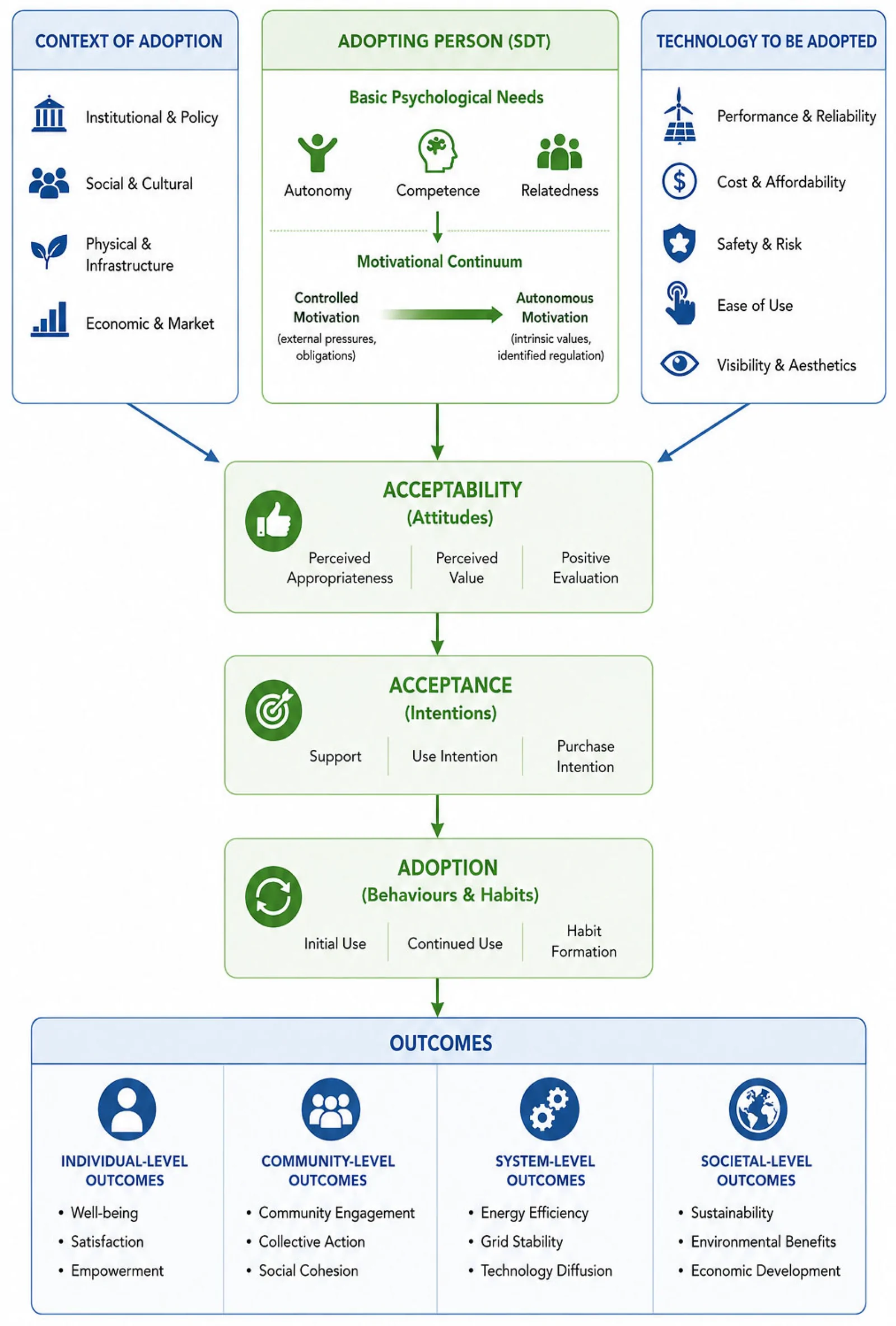
A SDT framework for the social acceptance of sustainable energy technologies based on the systematic review approach, according to the i-SETA model (Bonaiuto et al., 2023, 2024).

The importance of institutional support is particularly evident in emerging digital and AI-supported energy systems. Several recent studies emphasize that governance structures, regulatory transparency, and ethical AI frameworks are essential for maintaining public trust and long-term participation in technologically advanced sustainability systems (Malann et al., 2026; A. P. Zhao et al., 2025). Without adequate institutional safeguards, users may perceive smart energy infrastructures as controlling, intrusive, or unfair, thereby weakening autonomous motivation and increasing resistance. Taken together, the reviewed literature demonstrates that SDT processes operate within complex social, technological, financial/economics and policy/institutional environments (Figure 4). Autonomous motivation and psychological need satisfaction are important predictors of sustainable technology acceptance, but their effects are moderated by contextual conditions such as trust, fairness, socio-economic resources, technological usability, and policy design; as well as some sociodemographic and cultural factors. Consequently, sustainable energy transitions require not only technological innovation but also supportive social, financial and institutional contexts capable of fostering meaningful motivational engagement and long-term public participation.

Building upon the theoretical foundations of the integrated Sustainable Energy Technology Acceptance (i-SETA) model (Bonaiuto et al., 2023, 2024), the conceptual framework proposed in Figure 4 constitutes an SDT-based extension of i-SETA, rather than a validation or direct reproduction of the original model. Whereas the i-SETA model integrates within the social-psychological determinants of sustainable energy technologies acceptance, cognitive, affective, and motivational aspects, the present framework specifically reorganises these determinants through the lens of SDT. In particular, it explicitly positions basic psychological need satisfaction (autonomy, competence, and relatedness) and the motivational continuum (autonomous versus controlled motivation) as the central psychological mechanisms linking contextual conditions and technology characteristics to acceptability, acceptance, adoption, and subsequent multi-level outcomes. By synthesising evidence across diverse sustainable energy technologies and socio-technical contexts, this framework provides an integrative theoretical perspective intended to guide future empirical research rather than to replace the original i-SETA model.

Future empirical research could validate the proposed framework using structural equation modelling (SEM) or partial least squares structural equation modelling (PLS-SEM) to examine the relationships among contextual conditions, technology characteristics, basic psychological need satisfaction, motivational regulation, and technology acceptance outcomes. Longitudinal designs could further investigate how autonomous motivation develops over time and contributes to sustained technology adoption, while cross-cultural comparative studies could examine whether these motivational pathways differ across socio-economic, institutional, and cultural contexts. Such empirical validation would help refine and extend the proposed conceptual framework.

## 4. Discussion

### 4.1. Theoretical Contributions

This systematic review makes several important theoretical contributions by substantially extending the application of SDT into the domain of sustainable energy transitions, smart energy systems, and technology acceptance. Although SDT has been widely applied in fields such as education, health, and organizational behaviour, its integration into the field of sustainable energy technologies has remained fragmented and conceptually dispersed.

By synthesising evidence across renewable energy adoption, smart grids, electric vehicles, decentralized storage systems, energy citizenship, gamification, and AI-enabled energy platforms, this review demonstrates that SDT offers largely coherent and versatile framework for understanding the motivational foundations of sustainable technology acceptance and long-term public engagement. More importantly, rather than merely summarising previous findings, this review integrates diverse empirical evidence into a unified conceptual perspective that links autonomous motivation, controlled motivation, basic psychological need satisfaction, perceived technological features and related beliefs, contextual moderators (perceived policy and economics beliefs), and technology acceptance outcomes. This integrative perspective provides the theoretical foundation for the conceptual framework proposed in Figure 4 and offers a basis for future empirical research.

Unlike previous reviews that primarily summarised SDT-related findings across individual sustainability contexts, the present review proposes an integrated conceptual framework that organises the existing evidence into a coherent explanatory model. Specifically, it synthesises the relationships among autonomous motivation, controlled motivation, basic psychological need satisfaction, beliefs about the technology and its context of acceptance, and sustainable technology acceptance outcomes. Rather than validating or reproducing the original i-SETA model, this framework constitutes an SDT-based extension: it reorganises the determinants identified by i-SETA around a motivational core, offering a more explicit account of why and how psychological need satisfaction and motivation quality shape technology acceptance, and identifying potential pathways for future empirical validation. More importantly, the present review advances SDT itself by demonstrating how its core motivational principles can be systematically applied across diverse sustainable energy and socio-technical contexts, thereby extending SDT from an individual motivational theory to a broader explanatory framework for understanding long-term sustainable energy transitions.

Building upon this integrated framework, the present review highlights several specific theoretical contributions. First, this review advances both technology acceptance research and the application of SDT by introducing a motivational and value-based perspective that complements traditional cognitive and affective acceptance models. Rather than revisiting the limitations of traditional technology acceptance models, the present review demonstrates how SDT complements these frameworks by explaining the motivational quality that underlies sustained behavioural engagement. Building upon this foundation, our review further integrates evidence showing how autonomous motivation, controlled motivation, and psychological need satisfaction jointly influence technology acceptance across diverse sustainable energy contexts.

Importantly, the reviewed literature suggests that sustainable energy transitions differ from many conventional technology-adoption contexts because they involve moral values, environmental identity, collective responsibility, and long-term behavioural commitment. In these settings, acceptance is not merely a utilitarian decision but also reflects individuals’ broader sense of self, citizenship, and participation in sustainability transitions. SDT therefore helps to explain how collective engagement and civic participation emerge within energy-transition processes, thereby demonstrating how individual motivational processes can be integrated with broader socio-technical transition frameworks. This synthesis represents one of the principal theoretical contributions of the present review.

Second, this review contributes to sustainability-transition theory by bridging micro-level social-psychological processes with macro-level socio-technical transformations. Existing sustainability-transition frameworks often emphasize technological innovation, governance structures, market diffusion, and institutional change (Köhler et al., 2019), but they pay comparatively less attention to motivational processes and psychological need satisfaction. The reviewed studies demonstrate that autonomy, competence, and relatedness operate as critical mechanisms influencing how individuals engage with decentralized energy systems, smart technologies, community participation, and energy governance processes. In this sense, SDT provides a psychologically grounded explanation for why some sustainability initiatives achieve meaningful public engagement, while other ones encounter resistance or superficial compliance. These findings also broaden the explanatory scope of SDT by demonstrating that psychological need satisfaction is relevant not only to individual behavioural regulation but also to collective participation and socio-technical transformation.

This contribution is especially relevant in the context of energy citizenship and participatory sustainability transitions. Several recent studies move beyond simple technology adoption and instead conceptualize citizens as active participants in energy governance, energy communities, and collective sustainability practices (Awopone et al., 2025; Brizga & Vijaikis, 2024; Vorobeva et al., 2026). The findings suggest that sustainable transitions are more likely to succeed when individuals perceive themselves as autonomous, competent, and socially connected participants within broader socio-technical systems. SDT therefore helps to explain how collective engagement and civic participation emerge within energy-transition processes, in order to boost SDT’s basic motivational pillars (competence, autonomy, relatedness).

Third, the review advances theoretical understanding of controlled motivation and motivational crowding within sustainability contexts. Traditional environmental policies frequently rely on financial incentives, regulatory mandates, or informational campaigns to encourage behavioural change. However, the reviewed studies consistently indicate that excessive reliance on external rewards may undermine intrinsic engagement and weaken long-term commitment (Awopone et al., 2025; Stauch & Gamma, 2020). At the same time, the findings reveal important contextual nuances: controlled motivation may still remain effective when under economically constrained settings, where financial barriers strongly shape adoption decisions (Al-Habaibeh et al., 2023; S. Wang et al., 2024). These findings contribute to SDT by demonstrating that the effects of external incentives are highly context-dependent and interact with broader socio-economic and institutional conditions. Accordingly, the present review extends existing SDT applications by highlighting the contextual conditions under which controlled motivation may either undermine or facilitate sustainable technology acceptance.

Fourth, this review extends SDT into emerging digital and AI-enabled sustainability systems. A particularly novel contribution of the reviewed literature is the increasing integration of SDT into smart grids, gamified energy-management systems, AI-powered energy platforms, and peer-to-peer energy trading environments (Zhang et al., 2025; Malann et al., 2026). These studies suggest that SDT is not limited to explaining traditional pro-environmental behavior, it is rather increasingly relevant for understanding human–technology interaction within digitally mediated sustainability infrastructures. In these contexts, autonomy concerns, algorithmic transparency, cognitive load, data vulnerability, and user empowerment become central determinants of acceptance and engagement. Consequently, SDT offers a valuable framework for examining the social-psychological dimensions of increasingly intelligent and automated energy systems. This finding also illustrates the adaptability of SDT to emerging technological environments that extend well beyond its traditional application domains.

Overall, the theoretical contribution of this review lies in demonstrating that motivation is not a peripheral factor but rather a central mechanism underlying sustainable technology acceptance, energy citizenship, and long-term sustainability transitions. The reviewed evidence suggests that successful sustainability transitions depend not only on technological efficiency or economic incentives but also on whether technologies, policies, and institutions support individuals’ psychological needs and foster meaningful motivational internalisation. Taken together, the present review synthesises the existing evidence and advances SDT by integrating motivational regulation, psychological need satisfaction, perceived technology’s features and contextual influences, and technology acceptance outcomes into a single conceptual framework. This framework extends previous SDT applications beyond traditional pro-environmental behaviour and provides a theoretically grounded foundation for future empirical testing across diverse sustainable energy contexts.

### 4.2. Integration with Environmental Psychology

The findings of this review appear to align with and offer extensions to several major theoretical traditions within environmental psychology, particularly those concerned with pro-environmental behaviour, environmental identity, social norms, and value-based decision-making. Integrating SDT with these frameworks provides a deeper understanding of the motivational mechanisms underlying sustainable technology acceptance and behavioural engagement.

One of the most important theoretical connections is with the Value-Belief-Norm (VBN) theory, which proposes that pro-environmental behaviour emerges through a chain linking personal values, environmental beliefs, awareness of consequences, and moral norms (Stern, 2000; Stern et al., 1999). The reviewed studies generally support this value-based perspective. Specifically, research shows that biospheric and altruistic values are associated with more autonomous forms of motivation and stronger support for sustainable energy technologies (De Groot & Steg, 2010; Legault et al., 2024). From an SDT perspective, these values become behaviourally influential when they are internalised into individuals’ sense of self through identified or integrated regulation. SDT therefore complements VBN theory by explaining how environmental values are transformed into sustained behavioural commitment through motivational internalisation processes.

The review also extends broader models of pro-environmental behaviour such as the Theory of Planned Behavior (TPB) (Ajzen, 1991). While TPB emphasizes attitudes, subjective norms, and perceived behavioural control, the reviewed studies suggest that these cognitive and social determinants are strongly shaped by the satisfaction or frustration of psychological needs. For example, social norms appear most effective when they support relatedness and collective identity rather than functioning as controlling social pressure (Brizga & Vijaikis, 2024; Tirotto & Pahl, 2023). Similarly, perceived behavioural control becomes psychologically meaningful when individuals experience competence and autonomy in relation to sustainable technologies. In this sense, SDT deepens TPB by clarifying the motivational quality underlying attitudes, norms, and behavioural intentions.

Another important contribution concerns environmental identity and energy citizenship. Environmental psychology has increasingly emphasized that sustainable behaviours are linked to individuals’ self-concepts and social identities (Molinario et al., 2020; van der Werff et al., 2013; Whitmarsh & O’Neill, 2010). The reviewed studies provide further support for this perspective. Research on energy citizenship, community participation, and decentralized energy systems consistently demonstrates that individuals are more willing to engage with sustainability transitions when environmental engagement becomes integrated into their broader sense of self and social belonging (Awopone et al., 2025; Brizga & Vijaikis, 2024). SDT contributes to this literature by explaining how relatedness, autonomy, and value internalisation facilitate the integration of environmental behaviours into personal and collective identities.

The review further highlights the growing relevance of environmental psychology in digitally mediated sustainability systems. Emerging technologies such as smart grids, AI-enabled energy management systems, gamified energy platforms, and peer-to-peer energy trading environments create new forms of human–technology interaction that cannot be fully explained by traditional environmental-behaviour models alone. The reviewed studies suggest that psychological factors such as trust, data vulnerability, cognitive load, algorithmic transparency, and perceived autonomy increasingly shape public responses to these technologies (Malann et al., 2026; Simon & Schweitzer, 2023). This indicates that environmental psychology must increasingly engage with digitalization, automation, and intelligent infrastructures as core components of sustainability transitions (e.g., De Dominicis et al., 2019).

Importantly, the findings also suggest that sustainable technology acceptance is deeply social and relational rather than purely individualistic. Community trust, procedural justice, social norms, and collective participation repeatedly emerge as critical moderators of motivation and engagement (Bögel et al., 2021; Vorobeva et al., 2026). These findings align closely with socio-environmental perspectives emphasizing that sustainability transitions involve collective practices, shared meanings, and institutional relationships rather than isolated consumer decisions. SDT enriches these perspectives by identifying the motivational bases and processes through which collective engagement and participation become psychologically meaningful.

Taken together, these theoretical connections suggest that SDT does not replace existing environmental psychology frameworks but rather extends and integrates them. By focusing on motivational quality, psychological need satisfaction, and internalisation processes, SDT provides a more integrated explanation of how environmental values, social norms, technological systems, and institutional contexts interact to shape sustainable behaviour and technology acceptance.

### 4.3. Practical Implications

The findings of this review offer several practical implications for policymakers, technology developers, engineers, communication practitioners, and sustainability stakeholders seeking to promote sustainable energy transitions. The reviewed evidence suggests that successful transitions require more than technological efficiency or economic incentives; they may also depend on whether sustainable systems support SDT’s basic pillars, namely individual’s autonomy, competence, and relatedness. These factors may contribute to the social acceptance process by fostering trust and meaningful public engagement.

For policymakers, the findings highlight the potential value of designing autonomy-supportive sustainability policies. The findings of the reviewed studies suggest that policies encouraging meaningful participation, procedural fairness, and autonomy support may contribute to greater public acceptance of sustainable energy technologies. Studies on decentralized energy systems, geothermal energy projects, and energy communities suggest that individuals are more willing to support sustainability initiatives when they perceive decision-making processes as fair, participatory, and respectful of community identities (Tirotto & Pahl, 2023; Vorobeva et al., 2026). Accordingly, policymakers may benefit from complementing one-directional information campaigns with participatory governance structures that encourage psychological ownership and collective engagement.

The findings also suggest that overreliance on financial incentives may produce limited or unstable behavioural effects. Although economic incentives remain important in financially constrained contexts (Al-Habaibeh et al., 2023; S. Wang et al., 2024), several studies demonstrate that excessive dependence on external rewards may crowd out intrinsic motivation and weaken long-term engagement (Awopone et al., 2025; Stauch & Gamma, 2020). Consequently, sustainability policies may be strengthened by balancing economic support with strategies that promote value internalisation, environmental identity, and community participation. Policies framed around autonomy, environmental responsibility, and collective benefits may therefore be more likely to support enduring behavioural change than purely financial or punitive interventions.

For engineers, technology developers, and energy-system designers, the review emphasizes the potential importance of user-centred and psychologically informed technological design. Across smart meters, AI-enabled systems, and decentralized energy technologies, users are more likely to engage with systems that are transparent, intuitive, and supportive of competence and autonomy (Koo & Chung, 2014; Thunshirn et al., 2025), as well as relatedness (De Dominicis et al., 2019). Conversely, excessive complexity, cognitive overload, opaque algorithms, and perceived surveillance may reduce trust and discourage engagement (Simon & Schweitzer, 2023). These findings suggest that technical performance alone is insufficient; sustainable technologies may also need to be social-psychologically accessible and empowering.

This issue is particularly important within emerging AI-supported energy systems and smart infrastructures. The reviewed studies highlight growing concerns regarding data privacy, algorithmic transparency, and psychological disempowerment within digitally mediated sustainability systems (Malann et al., 2026; Zhang et al., 2025). Developers may therefore consider prioritizing explainable AI, transparent data governance, and user control mechanisms to ensure that automation enhances rather than undermines users’ sense of autonomy and competence, as well as their social relations. In this context, ethical and psychologically informed design may play a critical role in supporting public acceptance of future smart energy infrastructures.

Communication practitioners and sustainability advocates can also draw useful insights from the reviewed literature. Communication strategies that rely exclusively on fear appeals, technical information, or financial savings may be less effective than approaches that emphasise environmental values, collective identity, autonomy, and social participation. Studies on energy citizenship and community engagement consistently show that people are more motivated when sustainable behaviours are connected to meaningful identities, shared goals, and collective benefits (Awopone et al., 2025; Brizga & Vijaikis, 2024). Consequently, sustainability communication may benefit from framing energy transitions not merely as technical adjustments, but also as opportunities for empowerment, participation, and social contribution (Agozie et al., 2023).

The findings further suggest that gamification and digital engagement strategies may offer promising tools for enhancing sustainable behaviour when designed appropriately. Gamified systems that support autonomy, competence, and relatedness through feedback, collaborative challenges, and community participation have the potential to increase engagement with energy-saving practices and smart energy systems (Jufri et al., 2026; Y. Wang et al., 2025). However, these strategies should avoid excessive competition, manipulation, or behavioural control, which may undermine intrinsic motivation over time.

Overall, the findings suggest that sustainable energy transitions may benefit from a holistic and social-psychologically informed approach integrating technological innovation, motivational support, social trust, participatory governance, and user-centred system design. Technologies and policies are more likely to achieve broader public acceptance when they support individuals as active participants in sustainability transitions, rather than treating them merely as passive consumers or targets of behavioural regulation.

### 4.4. Limitations

Despite its theoretical and practical contributions, this review is subject to several limitations that should be acknowledged. First, although the methodological quality of eligible empirical studies was assessed using the MMAT, the included literature comprised diverse study designs. Theoretical studies and simulation-based studies could not be appraised using the MMAT because their methodological characteristics fall outside the scope of this appraisal tool. Therefore, variations in study designs and methodological characteristics should be considered when interpreting the synthesized findings. In addition, although the reviewed literature spans a broad range of sustainable energy technologies and related socio-technical contexts, the overall evidence base remains methodologically uneven. A large proportion of the included studies employ cross-sectional survey designs using self-reported behavioural intentions rather than actual long-term behavioural outcomes. While these studies provide valuable insights into motivational mechanisms, they limit the ability to establish causal relationships and to examine how motivation, engagement, and technology acceptance evolve over time. This limitation is particularly important because sustainability transitions are inherently dynamic and long-term processes, involving gradual behavioural adaptation, technological learning, and changing social norms. Only a relatively small number of studies adopted longitudinal, experimental, or mixed-methods approaches capable of capturing these temporal dynamics (e.g., Peng & Klöckner, 2026; Stauch & Gamma, 2020).

Second, although the reviewed studies demonstrate increasing geographical diversity, important regional and cultural imbalances remain. European contexts dominate the literature, particularly studies on smart grids, decentralized energy systems, energy citizenship, and community participation. By contrast, evidence from Africa, Latin America, the Middle East, and parts of Asia remains comparatively limited. Furthermore, studies conducted in developing economies often focus more strongly on financial barriers, infrastructural limitations, and policy constraints; whereas studies from European contexts more frequently emphasize autonomy, participation, and environmental identity. These differences suggest that SDT processes may operate differently across socio-economic and cultural settings, particularly regarding the relative importance of autonomy, competence, social relations, and external incentives. Consequently, the generalisability of existing findings across diverse global contexts remains uncertain.

Third, the reviewed literature is highly heterogeneous in terms of theoretical framing, methodological design, and technological focus. The dataset includes quantitative surveys, qualitative interviews, experiments, conceptual analyses, systematic reviews, and technical optimization studies integrating SDT into AI-driven and gamified energy systems. While this diversity enriches the review, it also creates challenges for synthesis and comparison. Different studies operationalize SDT constructs in different ways, with some focusing primarily on intrinsic motivation; others emphasizing identified regulation or psychological need satisfaction; and still others integrating SDT with frameworks such as TAM, TPB, VBN, COR theory, or gamification models. As a result, direct comparison across studies is not always straightforward. Moreover, synthesising conceptual and empirical studies within a single thematic framework inevitably involves a degree of interpretative judgement. Accordingly, the integrative framework proposed in this review should be regarded as a theoretically informed synthesis of the available evidence rather than a definitive or empirically validated model.

The diversity of technological contexts further complicates synthesis. The reviewed studies examine highly varied domains, including solar photovoltaics, smart meters, geothermal energy, electric vehicles, peer-to-peer energy trading systems, AI-supported energy platforms, gamified systems, and energy citizenship initiatives. Although common motivational patterns emerge across these contexts, the psychological processes underlying acceptance may differ substantially depending on technological complexity, automation, infrastructural conditions, and levels of public participation. For example, concerns related to algorithmic transparency and data vulnerability are highly relevant in AI-enabled smart systems, but less central in studies of residential solar adoption or household conservation behaviour.

Fourth, the review reveals that the current literature still places greater emphasis on technology adoption intentions than on sustained long-term behavioural engagement and post-adoption experiences. Although several studies examine energy citizenship, behavioural persistence, and community participation, many studies remain focused on initial acceptance or behavioural intention. Consequently, less is known about how autonomous motivation is maintained—or weakened—over time as individuals interact with increasingly digitalized and automated sustainability systems.

Another important limitation concerns the emerging nature of research on AI-supported and gamified energy systems. Several recent studies integrate SDT into smart grids, peer-to-peer trading, and AI-driven energy management platforms, but many of these studies remain conceptual, simulation-based, or technically oriented rather than empirically validated in real-world settings (Zhang et al., 2025; A. P. Zhao et al., 2025). Consequently, evidence regarding how users psychologically experience highly automated sustainability systems remains relatively limited.

Finally, the review relies exclusively on peer-reviewed English-language publications indexed in major academic databases. This may introduce publication bias, language bias, and disciplinary bias, as studies reporting non-significant findings, locally published research, or grey literature may be underrepresented. In addition, because sustainability transitions are rapidly evolving, some emerging technological and policy developments may not yet be fully reflected within the academic literature.

### 4.5. Future Research

Building on the limitations identified above, several important directions for future research can be proposed. First, there is a clear need for more longitudinal and process-oriented research examining how motivation, behavioural engagement, and technology acceptance evolve over time. Sustainability transitions involve ongoing adaptation rather than one-time adoption decisions, yet much of the current literature remains cross-sectional. Future studies should therefore investigate how autonomous and controlled motivation change throughout different stages of technology adoption, continued use, behavioural retention, and disengagement. Longitudinal approaches may be especially valuable for understanding behavioural persistence in contexts such as smart energy systems, energy-saving practices, electric vehicle use, and energy citizenship.

Second, future research should place greater emphasis on real-world behavioural outcomes rather than relying primarily on self-reported intentions. Many reviewed studies examine attitudes, intentions, or willingness to adopt technologies, but fewer assess actual long-term behaviours, sustained participation, or measurable energy outcomes. Combining behavioural data, smart-meter records, digital platform analytics, and longitudinal tracking could provide a more comprehensive understanding of how SDT processes influence real-world sustainability practices over time.

Third, additional cross-cultural and comparative research is urgently needed. The reviewed literature suggests that motivational processes interact with socio-economic conditions, institutional arrangements, energy infrastructures, and cultural values. Future studies should therefore examine how SDT constructs operate across different political systems, levels of economic development, and cultural contexts. Comparative research may help to clarify whether psychological needs such as autonomy and relatedness function differently in collectivistic versus individualistic cultures or in contexts characterized by differing levels of energy insecurity, digitalization, and institutional trust.

Fourth, future research should further investigate the growing role of AI, automation, and digitalization in sustainable energy systems. Emerging technologies such as AI-powered energy management systems, smart grids, algorithmic demand response systems, and peer-to-peer energy trading platforms fundamentally reshape human–technology interaction. The reviewed studies suggest that these systems introduce novel psychological concerns related to data vulnerability, surveillance, cognitive overload, algorithmic opacity, and perceived loss of autonomy (Malann et al., 2026; Simon & Schweitzer, 2023). Future research should therefore examine how SDT can inform ethical AI design, explainable automation, user empowerment, and psychologically sustainable digital infrastructures.

Relatedly, the rapid growth of gamified sustainability systems represents another important research direction. Several recent studies suggest that gamification strategies may strengthen engagement by supporting autonomy, competence, and relatedness, yet empirical evidence regarding their long-term effectiveness remains limited. Future research should investigate whether gamified interventions produce durable behavioural internalisation or whether they risk becoming externally controlling systems that eventually undermine intrinsic motivation.

Another promising direction involves expanding research on energy citizenship, collective participation, and community-based sustainability transitions. Much of the existing literature still conceptualizes individuals primarily as consumers or adopters of technologies. However, recent studies increasingly emphasize participatory governance, collective engagement, and community energy systems. Future research should therefore examine how social identity, collective efficacy, procedural justice, and relatedness interact with SDT processes in shaping collaborative sustainability practices and civic participation.

Methodologically, interdisciplinary and mixed-methods approaches could further advance research in this area. Combining quantitative modelling with qualitative interviews, ethnographic observation, behavioural tracking, and experimental interventions may provide richer insights into how individuals psychologically experience sustainability transitions in everyday life. In particular, qualitative approaches could deepen understanding of how users interpret autonomy, trust, control, and participation within increasingly complex digital energy systems.

Finally, future research should further integrate SDT with complementary theoretical frameworks such as environmental identity theory, Value-Belief-Norm theory, socio-technical transition theory, behavioural economics, and human–computer interaction research. Given the growing complexity of sustainable energy systems, no single theoretical framework is likely to fully explain technology acceptance and behavioural engagement. More integrative and multidisciplinary models may therefore provide a comprehensive understanding by devoting a triadic attention to both the technology, the person, and their surrounding context. This means that i) certain perceived features of the technology to be adopted, ii) various perceived contextual factors (both economics and political factors) framing the adoption process, and iii) specific personal factors of the adopting person (individual social-psychological variables), all jointly shape sustainable energy transitions (Bonaiuto et al., 2023, 2024; Dessi et al., 2022; Milani et al., 2024).

## 5. Conclusions

This systematic review demonstrates that SDT provides a powerful and highly relevant framework for understanding the social acceptance of sustainable energy technologies. Across studies examining renewable energy adoption, smart grids, electric vehicles, decentralized energy storage systems, energy citizenship initiatives, gamified sustainability platforms, and AI-enabled energy systems, the reviewed evidence suggests that engagement is shaped not only by technological efficiency or economic incentives but also by the quality of individuals’ motivation and their psychological experiences.

A central finding of the review is that autonomous motivation plays a particularly important role in fostering meaningful and enduring support for sustainability transitions. Individuals are more likely to adopt, support, and sustain engagement with sustainable technologies when environmental behaviours are aligned with their personal values, identities, and sense of social responsibility. In contrast, approaches relying primarily on external pressure, financial incentives, or coercive regulation often produce weaker internalisation and less stable long-term engagement. These findings support SDT’s distinction between autonomous and controlled forms of motivation and highlight the importance of motivational quality in shaping sustainable behaviour in the energy sector too.

The review further demonstrates that the satisfaction of the three basic psychological needs—autonomy, competence, and relatedness—is central to sustainable energy technology acceptance. Technologies and policies are more likely to succeed when individuals experience meaningful choice and participation, feel capable of understanding and using systems effectively, and perceive themselves as connected to broader social and environmental communities. Conversely, technologies that generate cognitive overload, algorithmic opacity, perceived surveillance, or psychological disempowerment may undermine trust and weaken engagement even when environmental attitudes are positive.

Importantly, the findings suggest that sustainable energy transitions are not solely technical or economic transformations but also deeply psychological and socio-cultural processes. Public engagement with renewable energy systems, smart infrastructures, and community-based energy initiatives depends heavily on trust, procedural fairness, social identity, institutional transparency, and opportunities for meaningful participation. In this sense, sustainability transitions increasingly require human-centred approaches that recognize citizens not merely as passive consumers but as active participants in socio-technical change.

The review also highlights the growing relevance of SDT within digitally mediated sustainability systems. Emerging AI-supported and gamified energy technologies create new forms of human–technology interaction that reshape experiences of autonomy, competence, social relatedness, thus impacting trust and behavioural engagement. Consequently, psychologically informed and ethically responsible system design may become increasingly important for ensuring public acceptance of future smart energy infrastructures.

Overall, this review demonstrates that technological advancement alone may be insufficient to achieve sustainable futures. Long-term sustainability transitions require motivationally supportive policies, social-psychologically empowering technologies, participatory governance structures, and socially meaningful forms of engagement that foster the internalisation of environmental values and identity, and collective responsibility. By integrating SDT into sustainability-transition research, this review provides a theoretically grounded and practically relevant framework for understanding how human motivation shapes the acceptance and successful implementation of sustainable energy technologies in an increasingly complex and digitalized world.

## Figures and Tables

**Table 1:** SDT-based motivational drivers across sustainable energy contexts.

Sustainable Energy Domains	Basic Psychological Needs	Motivational Types	Supporting Studies
Smart grids & digital metering systems	Autonomy; competence	Autonomous motivation	Wunderlich et al. (2013); Joachain and Klopfert (2014); Koo and Chung (2014); Murtagh et al. (2014); Wunderlich et al. (2019); Simon and Schweitzer (2023); Thunshirn et al. (2025); Y. Wang et al. (2025); Zhang et al. (2025)
Community initiatives & energy citizenship	Autonomy; relatedness	Autonomous motivation; extrinsic motivation	Flostrand et al. (2019); Stauch and Gamma (2020); Bögel et al. (2021); Brambati et al. (2022); Tirotto and Pahl (2023); Awopone et al. (2025); Vorobeva et al. (2026)
Electric vehicles & alternative mobility	Autonomy; competence; relatedness	Autonomous motivation; extrinsic motivation	De Groot and Steg (2010); Sugihara and Hardman (2022); Mohamad et al. (2024); H. Zhao et al. (2024); Dias et al. (2025); A. P. Zhao et al. (2025)
Household microgeneration & technology adoption	Autonomy (autarky); competence; relatedness	Autonomous motivation; extrinsic motivation	Ecker et al. (2017); Al-Marri et al. (2018); Ecker et al. (2018); Al-Habaibeh et al. (2023); Chikandiwa and Mutambara (2023); Aguayo-Mendoza et al. (2024); Brizga and Vijaikis (2024); Legault et al. (2024); Nagarajan et al. (2025); S. Wang et al. (2024); Chanda et al. (2026); Peng and Klöckner (2026)

## Data Availability

No new data were created or analyzed in this study. Data sharing is not applicable to this article.

## Appendix A. Overview of All Studies Included in the Review

**Author(s) & Year**	**Country**	**Title**	**Study Design**	**Sample & Size**	**Technology Context**	**Main Results**	**Journal**	**MMAT Quality Rating**
De Groot and Steg (2010)	Netherlands	Relationships between value orientations, self-determined motivational types and pro-environmental behavioural intentions	Cross-sectional survey	Study 1: N = 304; Study 2: N = 520 (undergraduate students)	Fuel efficiency/low-emission vehicle	Values (especially biospheric) were generally stronger predictors of pro-environmental intentions than self-determined motivational types. Altruistic and biospheric values were positively related to more self-determined motivations, while egoistic values were linked to less self-determined motivations.	Journal of Environmental Psychology	High (5)
Wunderlich et al. (2013)	Germany	The impact of endogenous motivations on adoption of IT-enabled services: The case of transformative services in the energy sector	Cross-sectional survey	N = 999 (462 users, 537 nonusers)	Home energy management services, smart metering	Internal (autonomous) motivation strongly predicts adoption intention; external motivation matters less. Users vs. nonusers differ in motivation patterns.	Journal of Service Research	High (4)
Joachain and Klopfert (2014)	Belgium	Smarter than metering? Coupling smart meters and complementary currencies to reinforce the motivation of households for energy savings	Conceptual analysis; policy design	No empirical sample	Smart meters, household energy saving	Using green points/rewards (not money) with smart meters strengthens autonomous motivation for energy saving better than financial incentives. Reward-based systems work best; hybrid designs are better than strict regulatory ones.	Ecological Economics	–
Koo and Chung (2014)	South Korea	Examining the eco-technological knowledge of smart green IT adoption behavior: A self-determination perspective	Cross-sectional survey	N = 100 (smart energy-saving device users)	Smart Green IT, energy-saving power management devices	Eco-technological knowledge boosts intrinsic and autonomous extrinsic motivation. Intrinsic motivation enhances all extrinsic motivations. Attitude is the strongest predictor of continued usage; social influence and external regulation also matter.	Technological Forecasting and Social Change	High (5)
Murtagh et al. (2014)	United Kingdom	20:60:20—Differences in energy behaviour and conservation between and within households with electricity monitors	Qualitative (semi-structured interviews + thematic analysis)	N = 21 households (IHD users)	In-home displays, smart meter feedback	Households fell into three types: Monitor Enthusiasts (20%), Aspiring Energy Savers (60%), and Energy Non-engaged (20%). Only 4 households continued active IHD use after 6+ months. Energy saving behaviours were influenced by social practices, household relationships, and financial motivations. Women played a key role in domestic energy use decisions.	PLOS ONE	High (5)
Ecker et al. (2017)	Germany, Switzerland	Promoting decentralized sustainable energy systems in different supply scenarios: The role of autarky aspiration	Mixed methods (survey + qualitative interviews)	Study 1: N = 168; Study 2: N = 13	Decentralized renewable energy, photovoltaic (PV) & battery storage	People prefer household-level energy autonomy for stronger independence, autonomy, control, and supply security; these psychological factors increase willingness to pay more than neighborhood or town-level systems. Journal Frontiers in Energy Research	Frontiers in Energy Research	High (5)
Al-Marri et al. (2018)	Qatar	An investigation into domestic energy consumption behaviour and public awareness of renewable energy in Qatar	Mixed methods (survey + expert interviews)	N = 410 consumers; 11 energy experts	Household energy, solar energy, energy efficiency	Free subsidized energy eliminates extrinsic motivation; education and environmental awareness are the most effective ways to promote energy conservation and solar adoption.	Journal Sustainable Cities and Society	Moderate (3)
Ecker et al. (2018)	Germany, Switzerland	Independence without control: Autarky outperforms autonomy benefits in private energy storage adoption	Cross-sectional survey; experimental design	Study 1: N = 240; Study 2: N = 249	PV, home battery storage, decentralized energy systems	Energy autarky strongly increases willingness to pay for storage systems; autonomy is desirable but does not drive payment. Autarky framing raises the value of self-generated energy and reduces peer-to-peer trading.	Energy Policy	High (5)
Flostrand et al. (2019)	Sweden	Better together—harnessing motivations for energy utility crowdsourcing activities	Conceptual study; qualitative interviews	N = 7 energy utility experts	Energy utility crowdsourcing, smart grid	Based on SDT, participation in energy utility crowdsourcing is driven by intrinsic and extrinsic motivations; five propositions are proposed to match different crowdsourcing activities with corresponding motivation mechanisms; reasonable incentive design and risk control are needed to ensure constructive participation.	Energy Research & Social Science	High (4)
Wunderlich et al. (2019)	Germany	Adoption of sustainable technologies: A mixed-methods study of German households	Mixed method (qualitative interviews + cross-sectional survey)	Study1: N = 24; Study: N = 930	Smart metering technology, smart grid, household sustainable energy adoption	Attitude, internal PLOC (perceived locus of causality), external PLOC, and inherent innovativeness positively predict adoption; privacy risk and age show negative effects; the STARS model is validated.	MIS Quarterly	High (5)
Stauch and Gamma (2020)	Switzerland	Cash vs. solar power: An experimental investigation of the remuneration-related design of community solar offerings	Between-subject experiment	N = 496 (valid N = 420)	Community solar, PV/BIPV, local renewable energy	Green customers prefer solar-only remuneration; financial rewards crowd out their intrinsic motivation. Default customers respond better to financial compensation. Interaction effect is significant.	Energy Policy	High (5)
Bögel et al. (2021)	Sweden, Portugal	What is needed for citizen-centered urban energy transitions: Insights on attitudes towards decentralized energy storage	Mixed methods (survey + stakeholder workshops)	Survey: Sweden N = 300, Portugal N = 65; Workshops: Sweden N = 18, Portugal N = 46	Decentralized energy storage, smart grid, community energy, building-level PV/battery	Citizens want autarky & autonomy but prefer low-effort automated systems; Swedes favor shared storage, Portuguese prefer individual systems; local social trust & sharing norms shape preferences.	Energy Policy	High (5)
Brambati et al. (2022)	Multi-country (17 European nations)	Predicting acceptance and adoption of renewable energy community solutions: the prosumer psychology	Cross-sectional survey	N = 212 respondents from 17 European countries	Renewable energy communities	Five latent psychological factors: concern about environmental issues, interest in energy sharing, concern on climate change, social influence, impact on bill cost. Three predictors of prosumer acceptance: attitude, economic incentive, and age (younger more likely).	Open Research Europe	High (5)
Sugihara and Hardman (2022)	USA	Electrifying California fleets: Investigating light-duty vehicle purchase decisions	Qualitative (semi-structured interviews + thematic analysis)	N = 23 fleet managers	Light-duty plug-in electric vehicles	Conventional vehicle purchases motivated by internalized extrinsic motivations (compatibility, standardization, price); PEV purchases driven by external regulations (sustainability goals, incentives, leadership). Barriers: infrastructure, model availability, range, costs, employee buy-in. Applies SDT to categorize motivation types along the extrinsic-intrinsic continuum.	Transportation Research Interdisciplinary Perspectives	High (5)
Al-Habaibeh et al. (2023)	Jordan	Solar energy in Jordan: Investigating challenges and opportunities of using domestic solar energy systems	Mixed methods (cross-sectional survey + qualitative free-text comments)	N = 366	Domestic solar PV, solar thermal, energy storage	High awareness but adoption limited by finance, space, and policy; users report strong satisfaction; extrinsic financial motivation dominates; government subsidies and low monthly payments are needed to boost uptake.	World Development Sustainability	Moderate (3)
Chikandiwa and Mutambara (2023)	South Africa	Green energy pathways: differing perspectives to green energy transitions among homeowners	Qualitative (semi-structured interviews + thematic analysis)	N = 19 university academics who are homeowners	Microgeneration technologies	Motivations: self-sufficiency, cost savings, environmental concern. Barriers: high initial costs, lack of information, perceived complexity of procurement process, security concerns, lack of support networks. Attitude-behaviour gap identified.	International Journal of Research in Business & Social Science	High (5)
Tirotto and Pahl (2023)	United Kingdom	Exploring the relationship between group-level autonomy and social acceptance of geothermal energy: The role of procedural justice climate, social identity violation, and collective self-determined motivation	Cross-sectional survey	N = 496 Cornish residents	Deep geothermal energy, community renewable energy	Group-level autonomy predicts acceptance via two paths: (1) procedural justice climate (moderated by social identity violation); (2) collective self-determined motivation. Social identity violation is the strongest negative predictor.	Psicologia Sociale	High (5)
Simon and Schweitzer (2023)	France	When smart meters backfire on energy transition internalization: Ethical electricity suppliers’ mitigation of consumer data vulnerability and attendant psychological disempowerment	Cross-sectional survey	N = 1121 homeowners with smart meters	Smart meters, smart grid, energy transition	Smart meter–induced data vulnerability reduces perceived competence and energy transition internalization; older consumers are more negatively affected; relational data transparency and fair exchange by suppliers mitigate these harms.	Technological Forecasting & Social Change	High (5)
Aguayo-Mendoza et al. (2024)	Europe & Latin America	Understanding the factors that affect households’ investment decisions required by the energy transition	Mixed methods (adapted Delphi expert panels + cross-sectional survey)	17 experts	Household energy transition	A 32-factor taxonomy identifies key drivers; competence & autonomy are prioritized over financial factors; regional & educational differences exist; policies should go beyond financial incentives.	PLOS ONE	High (4)
Brizga and Vijaikis (2024)	Latvia	Energy Citizenship: Revealing the intrinsic motivational factors suggested by self-determination theory	Cross-sectional survey	N = 749	Energy citizenship, energy transition	Intrinsic (personal & social) motivations significantly predict energy citizenship; extrinsic (financial/practical) motivations are non-significant or negative; energy citizenship positively predicts future willingness to participate.	Sustainability	High (4)
Legault et al. (2024)	USA	Pro-environmental, prosocial, pro-self, or does it depend? A more nuanced understanding of the motivations underlying residential solar panel adoption	Cross-sectional survey	N = 824 homeowners (214 adopters, 610 non-adopters)	Residential rooftop solar PV adoption	Autonomous pro-environmental motivation predicts solar adoption across demographics; controlled motivation predicts adoption only for younger/higher-income/less-educated homeowners; altruism predicts adoption for older households but negatively for younger; autonomous motivation predicts adoption for Democrats but not Republicans.	Energy Research & Social Science	High (5)
Mohamad et al. (2024)	Malaysia, Indonesia	A proposed model of electric vehicles user acceptance in developing countries: Mediating role of technophilia	Conceptual model	Planned N = 400 (200 each country)	Electric vehicle (EV)	Charging infrastructure, environmental/social values, and EV performance positively predict EV acceptance; technophilia mediates all these relationships; grounded in SDT (autonomy, competence, relatedness) and COR theory.	Global Business and Management Research: An International Journal	–
Nagarajan et al. (2025)	India	Distinct impacts of frugality on the intention to embrace energy-efficient and energy-generating products	Cross-sectional survey	N = 413 households	Energy-efficient and energy-generating products	Involuntary frugality (price sensitivity) positively correlates with energy-efficient product adoption but negatively with energy-generating product adoption. Deliberate frugality positively impacts both. Motivation to save fully mediates deliberate frugality → both product types, and partially mediates involuntary frugality → energy-efficient products. Draws on SDT (autonomy, competence, relatedness) to explain consumer motivation.	International Journal of Energy Sector Management	High (5)
S. Wang et al. (2024)	China	Does residential photovoltaic installation matter for coal-to-electricity transition? Evidence from self-determination theory perspective	Cross-sectional survey	N = 1136 rural residents (PV: 701; non-PV: 435)	Residential PV systems; coal-to-electricity transition	PV installation enhances energy autonomy, perceptual competence, and ownership consciousness, which mediate willingness to transition from coal to electricity. Economic incentives are more influential than environmental campaigns.	Current Psychology	High (5)
H. Zhao et al. (2024)	China	The influence of psychological factors on consumer purchase intention for electric vehicles: Case study from China: integrating the necessary condition analysis methodology from the perspective of self-determination theory	Cross-sectional survey	N = 478 valid respondents (non-EV owners)	EV	Psychological factors (autonomy, relatedness, competence) positively influence innovative consumption, which mediates EV purchase intention. Driving experience moderates the PEA→innovative consumption path. NCA identified necessary conditions for EPI.	World Electric Vehicle Journal	High (5)
Awopone et al. (2025)	Northern Ghana	Energy citizenship in northern Ghana: Drivers of community engagement in the sustainable energy transition	Cross-sectional survey	N = 678 rural residents	Community renewable energy, energy citizenship	Perceptions, motivation, and perceived benefits significantly boost community engagement; policy awareness has no significant effect. Perceived benefits strengthen the perception–engagement link but weaken the motivation–engagement link (motivational crowding-out).	Utilities Policy	High (4)
Dias et al. (2025)	India	Motivation continuum and its effect on electric vehicle acceptance in India	Cross-sectional survey	N = 351	EV, electric two-wheelers (E2W)	External, identified, and integrated motivation significantly predict EV adoption intention; PU, PEOU, introjected, and intrinsic motivation are non-significant. Gender moderates the effects of external, introjected, identified, and integrated motivation on BI. Extends TAM with SDT/Organismic Integration Theory (OIT).	Journal of Economic and Administrative Sciences	Moderate (3)
Thunshirn et al. (2025)	Austria	A qualitative analysis of consumer motivations and barriers towards active smart meter utilization	Qualitative semi-structured interviews	N = 26 residents	Smart meter	High willingness to use smart meters but low awareness of functions; key motivations: self-determination (autonomy/control), cost savings, pro-environmental values; key barriers: information deficit, cognitive load, risk aversion, social conflicts, perceived uselessness; privacy concerns are minor.	Energy Policy	High (5)
Y. Wang et al. (2025)	Not specified	Enhancing peer-to-peer energy trading in Integrated Energy Systems: Gamified engagement strategies and differentiable robust optimization	Technical paper (optimization framework case study)	Simulation: 100 residential prosumers; 20 commercial entities; 5 industrial loads	P2P energy trading; integrated energy systems	Framework integrates SDT (autonomy, competence, relatedness) for user engagement, DRO for uncertainty handling, and gamification (rewards, challenges, leaderboards). Case study shows 15% cost reduction, 20% carbon emission reduction, 25% increase in user engagement compared to conventional strategies.	Energy Reports	–
Zhang et al. (2025)	Not specified	Self-determination theory and deep reinforcement learning for personalized energy trading in smart grid	Technical paper (optimization framework simulation)	Simulation: Austin real-world data (2018)	P2P energy trading, home energy management (HEM), ESS, PV	Proposes leader-follower framework integrating SDT (autonomy, competence, relatedness) with DRL. Quantifies extrinsic (economic, comfort) and intrinsic (self-sufficiency, environmental) motivations via QoES matrix. Numerical results show proposed model outperforms standard DRL and optimization baselines.	IEEE Transactions on Systems, Man, and Cybernetics: Systems	–
A. P. Zhao et al. (2025)	Not specified	Psychological insights for electric vehicles	Technical paper (optimization framework case study)	Simulation: 10,000 EVs (25 clusters)	Vehicle-to-Grid systems, EV integration, renewable energy	Proposes interdisciplinary framework combining SDT (autonomy, competence, relatedness) with differentiable DRO for V2G optimization. Gamification and community-based clustering foster user engagement. Case study shows: 20% increase in participation rates, 25% reduction in peak grid dependency, 15% reduction in renewable curtailment.	International Journal of Electrical Power and Energy Systems	–
Chanda et al. (2026)	Bangladesh	From Conventional Fuels to Green Energy: Exploring Farmers’ Moral Commitment to Solar Photovoltaics Adoption	Cross-sectional survey	N = 356 farmers (PV nonadopters)	Solar PV	Awareness of consequences influences ascription of responsibility and intrinsic motivation, but not personal norms. Intrinsic motivation significantly affects personal norms and adoption intention. Personal norms positively predict adoption intention. GSI does not affect personal norms or adoption intention. fsQCA reveals nine configurations; intrinsic motivation emerges as necessary condition.	Sustainable Development	High (4)
Peng and Klöckner (2026)	Norway	Drivers and barriers to energy-saving behaviour formation and retention in response to extreme events: insights from the energy crisis	Longitudinal survey	Wave 1: N = 3514; Wave 2: N = 2289 households	Household energy-saving behaviours, energy crisis response	88% engaged in ≥1 energy-saving behaviour by May 2023; 65% retained behaviours after 6 months. Drivers: age, residential tenure, northern region, district heating. Barriers: larger households, comfort discomfort, affordability. Females and new heating system adopters more likely to sustain changes.	Sustainability Science	High (5)
Vorobeva et al. (2026)	Netherlands, Spain, Italy	Bridging individual and collective action: Advancing energy community participation through demand response tools	Cross-sectional survey	N = 1807 energy consumers (solar experience)	Demand response, energy communities	Intrinsic motivation, identified regulation, external regulation, and trust positively influence behavioral intention. Behavioral intention and DRT use predict EC participation (spillover effect). Personal ecological norms moderate key relationships. For low-eco-norm individuals, DRT use has stronger impact on EC participation.	Energy Reports	High (4)

## References

[B1-behavsci-16-01343] Agozie D. Q., Afful-Dadzie A., Gyamfi B. A., Bekun F. V. (2023). Does psychological empowerment improve renewable energy technology acceptance and recommendation? Evidence from 17 rural communities. Renewable Energy.

[B2-behavsci-16-01343] Aguayo-Mendoza A., Irizar-Arrieta A., Casado-Mansilla D., Borges C. E. (2024). Understanding the factors that affect households’ investment decisions required by the energy transition. PLoS ONE.

[B3-behavsci-16-01343] Ajzen I. (1991). The theory of planned behavior. Organizational Behavior and Human Decision Processes.

[B4-behavsci-16-01343] Alberts L., Lyngs U., Lukoff K. (2026). Designing for sustained motivation: A review of self-determination theory in behaviour change technologies. Interacting with Computers.

[B5-behavsci-16-01343] Al-Habaibeh A., Moh’d B. A. H., Massoud H., Nweke O. B., Al Takrouri M., Badr B. E. (2023). Solar energy in Jordan: Investigating challenges and opportunities of using domestic solar energy systems. World Development Sustainability.

[B6-behavsci-16-01343] Al-Marri W., Al-Habaibeh A., Watkins M. (2018). An investigation into domestic energy consumption behaviour and public awareness of renewable energy in Qatar. Sustainable Cities and Society.

[B7-behavsci-16-01343] Ariccio S., Lema-Blanco I., Bonaiuto M. (2021). Place attachment satisfies psychological needs in the context of environmental risk coping: Experimental evidence of a link between self-determination theory and person-place relationship effects. Journal of Environmental Psychology.

[B8-behavsci-16-01343] Ariccio S., Mosca O., Dessi F., Fornara F., Bonaiuto M. (2025). Cross-validation of the Biofuels Beliefs Scale (BBS) on a European sample: A tool to measure the perception of the technological and contextual features of biofuels. Technology in Society.

[B9-behavsci-16-01343] Awopone A. K., Amidu A. L. A., Antwi S. H., Ayambire P. N., Prempeh I. (2025). Energy citizenship in northern Ghana: Drivers of community engagement in the sustainable energy transitions. Utilities Policy.

[B10-behavsci-16-01343] Bonaiuto M., Dessi F., Ariccio S., Garcia-Mira R., Schweizer-Ries P., García-Fontán C. (2023). Acceptability, acceptance, and adoption of renewable and sustainable energy technologies. Sustainability and ecological transition in the post-COVID era: Challenges and opportunities in the face of climate change and energy transition.

[B11-behavsci-16-01343] Bonaiuto M., Mosca O., Milani A., Ariccio S., Dessi F., Fornara F. (2024). Beliefs about technological and con-textual features drive biofuels’ social acceptance. Renewable and Sustainable Energy Reviews.

[B12-behavsci-16-01343] Booth A., Sutton A., Papaioannou D. (2016). Systematic approaches to a successful literature review.

[B13-behavsci-16-01343] Bögel P. M., Upham P., Shahrokni H., Kordas O. (2021). What is needed for citizen-centered urban energy transitions: Insights on attitudes towards decentralized energy storage. Energy Policy.

[B14-behavsci-16-01343] Brambati F., Ruscio D., Biassoni F., Hueting R., Tedeschi A. (2022). Predicting acceptance and adoption of renewable energy community solutions: The prosumer psychology. Open Research Europe.

[B15-behavsci-16-01343] Braun V., Clarke V. (2006). Using thematic analysis in psychology. Qualitative Research in Psychology.

[B16-behavsci-16-01343] Brizga J., Vijaikis A. (2024). Energy citizenship: Revealing the intrinsic motivational factors suggested by self-determination theory. Sustainability.

[B17-behavsci-16-01343] Chanda R. C., Vafaei-Zadeh A., Hanifah H., Nikbin D., Sufian M. A. (2026). From conventional fuels to green energy: Exploring farmers’ moral commitment to solar photovoltaics adoption. Sustainable Development.

[B18-behavsci-16-01343] Chikandiwa C. T., Mutambara E. (2023). Green energy pathways: Differing perspectives to green energy transitions among homeowners. International Journal of Research in Business & Social Science.

[B19-behavsci-16-01343] Clarke V., Braun V. (2017). Thematic analysis. The Journal of Positive Psychology.

[B20-behavsci-16-01343] Claudy M. C., Peterson M., O’driscoll A. (2013). Understanding the attitude-behavior gap for renewable energy systems using behavioral reasoning theory. Journal of Macromarketing.

[B21-behavsci-16-01343] Cooke A. N., Fielding K. S., Louis W. R. (2016). Environmentally active people: The role of autonomy, relatedness, competence and self-determined motivation. Environmental Education Research.

[B22-behavsci-16-01343] Davis F. D. (1989). Perceived usefulness, perceived ease of use, and user acceptance of information technology. MIS Quarterly.

[B23-behavsci-16-01343] Deci E. L., Olafsen A. H., Ryan R. M. (2017). Self-determination theory in work organizations: The state of a science. Annual Review of Organizational Psychology and Organizational Behavior.

[B24-behavsci-16-01343] Deci E. L., Ryan R. M. (2000). The “what” and “why” of goal pursuits: Human needs and the self-determination of behavior. Psychological Inquiry.

[B25-behavsci-16-01343] De Dominicis S., Sokoloski R., Jaeger C. M., Schultz P. W. (2019). Making the smart meter social promotes long-term energy conservation. Palgrave Communications.

[B26-behavsci-16-01343] De Groot J. I., Steg L. (2010). Relationships between value orientations, self-determined motivational types and pro-environmental behavioural intentions. Journal of Environmental Psychology.

[B27-behavsci-16-01343] Dessi F., Ariccio S., Albers T., Alves S., Ludovico N., Bonaiuto M. (2022). Sustainable technology acceptability: Mapping technological, contextual, and social-psychological determinants of EU stakeholders’ biofuel acceptance. Renewable and Sustainable Energy Reviews.

[B28-behavsci-16-01343] Devine-Wright P. (2009). Rethinking NIMBYism: The role of place attachment and place identity in explaining place-protective action. Journal of Community & Applied Social Psychology.

[B29-behavsci-16-01343] Dias S., Davidson B. G. J., Chully A. A., Pendse P. H. (2025). Motivation continuum and its effect on electric vehicle acceptance in India. Journal of Economic and Administrative Sciences.

[B30-behavsci-16-01343] Ecker F., Hahnel U. J., Spada H. (2017). Promoting decentralized sustainable energy systems in different supply scenarios: The role of autarky aspiration. Frontiers in Energy Research.

[B31-behavsci-16-01343] Ecker F., Spada H., Hahnel U. J. (2018). Independence without control: Autarky outperforms autonomy benefits in the adoption of private energy storage systems. Energy Policy.

[B32-behavsci-16-01343] Flostrand A., Eriksson T., Brown T. E. (2019). Better together—Harnessing motivations for energy utility crowdsourcing activities. Energy Research & Social Science.

[B33-behavsci-16-01343] Gusenbauer M., Haddaway N. R. (2020). Which academic search systems are suitable for systematic reviews or meta-analyses? Evaluating retrieval qualities of Google Scholar, PubMed, and 26 other resources. Research Synthesis Methods.

[B34-behavsci-16-01343] Hobfoll S. E. (1989). Conservation of resources: A new attempt at conceptualizing stress. American Psychologist.

[B35-behavsci-16-01343] Hobfoll S. E., Halbesleben J., Neveu J. P., Westman M. (2018). Conservation of resources in the organizational context: The reality of resources and their consequences. Annual Review of Organizational Psychology and Organizational Behavior.

[B36-behavsci-16-01343] Hong Q. N., Fàbregues S., Bartlett G., Boardman F., Cargo M., Dagenais P., Gagnon M. P., Griffiths F., Nicolau B., O’Cathain A., Rousseau M. C., Vedel I., Pluye P. (2018). The Mixed Methods Appraisal Tool (MMAT) version 2018 for information professionals and researchers. Education for Information.

[B37-behavsci-16-01343] Intergovernmental Panel on Climate Change (IPCC) (2023). Climate change 2023: Synthesis report.

[B38-behavsci-16-01343] International Energy Agency (IEA) (2022). World energy outlook 2022.

[B39-behavsci-16-01343] Jabbour Al Maalouf N., Sayegh E., Inati D., Sarkis N. (2024). Consumer motivations for solar energy adoption in economically challenged regions. Sustainability.

[B40-behavsci-16-01343] Joachain H., Klopfert F. (2014). Smarter than metering? Coupling smart meters and complementary currencies to reinforce the motivation of households for energy savings. Ecological Economics.

[B41-behavsci-16-01343] Jufri K. S., Darhamsyah D., Tumpu M. (2026). Integrating gamification into ecological waste management systems: A narrative review for sustainable energy and environmental transition. Ecological Engineering & Environmental Technology.

[B42-behavsci-16-01343] Karbo R. T. V., Frewer L. J., Areal F., Jones G., Nurudeen S. (2024). A systematic review of the efficacy of theories used to understand farmers’ technology adoption behavior in lower-to-middle-income countries. Development Studies Research.

[B43-behavsci-16-01343] Koo C., Chung N. (2014). Examining the eco-technological knowledge of Smart Green IT adoption behavior: A self-determination perspective. Technological Forecasting and Social Change.

[B44-behavsci-16-01343] Köhler J., Geels F. W., Kern F., Markard J., Onsongo E., Wieczorek A., Alkemade F., Avelino F., Bergek A., Boons F., Fünfschilling L., Hess D., Holtz G., Hyysalo S., Jenkins K., Kivimaa P., Martiskainen M., McMeekin A., Mühlemeier M. S., Wells P. (2019). An agenda for sustainability transitions research: State of the art and future directions. Environmental Innovation and Societal Transitions.

[B45-behavsci-16-01343] Legault L., Bird S., Heintzelman M. D. (2024). Pro-environmental, prosocial, pro-self, or does it depend? A more nuanced understanding of the motivations underlying residential solar panel adoption. Energy Research & Social Science.

[B46-behavsci-16-01343] Malann D. C., Ogungbemi A. T., Edo G. I., Ali A. B., Jikah A. N., Yousif E., Francis U., Igbuku U. A., Oghroro E. E. A., Essaghah A. E. A., Ahmed D. S., Aliyu M. R., Umar H., Alamiery A. A. (2026). Designing AI-powered user-centric platforms for energy consumption and carbon footprint reduction. Sustainable Chemistry One World.

[B47-behavsci-16-01343] Milani A., Bonaiuto M., Packard C. D., Schultz P. W. (2026a). When norms surprise: The emotional power of descriptive norms in promoting sustainable energy engagement. Journal of Environmental Psychology.

[B48-behavsci-16-01343] Milani A., Dessi F., Bonaiuto M. (2024). A meta-analysis on the drivers and barriers to the social acceptance of renewable and sustainable energy technologies. Energy Research & Social Science.

[B49-behavsci-16-01343] Milani A., Dessi F., Bonaiuto M. (2026b). Why do people embrace renewable and sustainable energy technologies for mitigation and geoengineering for adaptation? Explore norms, emotions, and attitudes driving social acceptance. Journal of Environmental Psychology.

[B50-behavsci-16-01343] Mohamad S. H., Hafiz K. A., Shukri S. M., Makmor N., Azman N., Adiati R. P. (2024). A proposed model of electric vehicles user acceptance in developing countries: Mediating role of technophilia. Global Business & Management Research.

[B51-behavsci-16-01343] Molinario E., Lorenzi C., Bartoccioni F., Perucchini P., Bobeth S., Colléony A., Diniz R., Eklund A., Jaeger C., Kibbe A., Richter I., Ruepert A., Sloot D., Udall A. M., Bonaiuto M. (2020). From childhood nature experiences to adult pro-environmental behaviors: An explanatory model of sustainable food consumption. Environmental Education Research.

[B52-behavsci-16-01343] Mosca O., Milani A., Fornara F., Manunza A., Krys K., Maricchiolo F. (2023). Basic psychological needs, good societal development and satisfaction with life: The mediating role of the environment. Sustainability.

[B53-behavsci-16-01343] Mura A. L., Insalata L. A., Bonaiuto M. (2024). My home is my new office: The relationship between environmental comfort, workplace attachment, and psychological needs in the context of remote working. Journal of Environmental Psychology.

[B54-behavsci-16-01343] Murtagh N., Gatersleben B., Uzzell D. (2014). 20:60:20-Differences in energy behaviour and conservation between and within households with electricity monitors. PLoS ONE.

[B55-behavsci-16-01343] Nagarajan C. D., Afjal M., Idroes G. M. (2025). Distinct impacts of frugality on the intention to embrace energy-efficient and energy-generating products. International Journal of Energy Sector Management.

[B56-behavsci-16-01343] Olanipekun A. O., Chan A. P., Xia B., Adedokun O. A. (2018). Applying the self-determination theory (SDT) to explain the levels of motivation for adopting green building. International Journal of Construction Management.

[B57-behavsci-16-01343] Page M. J., McKenzie J. E., Bossuyt P. M., Boutron I., Hoffmann T. C., Mulrow C. D., Shamseer L., Tetzlaff J. M., Akl E. A., Brennan S. E., Chou R., Glanville J., Grimshaw J. M., Hróbjartsson A., Lalu M. M., Li T., Loder E. W., Mayo-Wilson E., McDonald S., Moher D. (2021). The PRISMA 2020 statement: An updated guideline for reporting systematic reviews. BMJ.

[B58-behavsci-16-01343] Pelletier L. G., Sharp E. (2008). Persuasive communication and proenvironmental behaviours: How message tailoring and message framing can improve the integration of behaviours through self-determined motivation. Canadian Psychology/Psychologie Canadienne.

[B59-behavsci-16-01343] Pelletier L. G., Tuson K. M., Green-Demers I., Noels K., Beaton A. M. (1998). Why are you doing things for the environment? The motivation toward the environment scale (MTES). Journal of Applied Social Psychology.

[B60-behavsci-16-01343] Peng Y., Klöckner C. A. (2026). Drivers and barriers to energy-saving behaviour formation and retention in response to extreme events: Insights from the energy crisis. Sustainability Science.

[B61-behavsci-16-01343] Perlaviciute G., Steg L. (2014). Contextual and psychological factors shaping evaluations and acceptability of energy alternatives: Integrated review and research agenda. Renewable and Sustainable Energy Reviews.

[B62-behavsci-16-01343] Petticrew M., Roberts H. (2006). Systematic reviews in the social sciences: A practical guide.

[B63-behavsci-16-01343] Ryan R. M., Deci E. L. (2000). Intrinsic and extrinsic motivations: Classic definitions and new directions. Contemporary Educational Psychology.

[B64-behavsci-16-01343] Ryan R. M., Deci E. L. (2024). Self-determination theory. Encyclopedia of quality of life and well-being research.

[B65-behavsci-16-01343] Simon F., Schweitzer V. (2023). When smart meters backfire on energy transition internalization: Ethical electricity suppliers’ mitigation of consumer data vulnerability and attendant psychological disempowerment. Technological Forecasting and Social Change.

[B66-behavsci-16-01343] Sintov N. D., Schultz P. W. (2015). Unlocking the potential of smart grid technologies with behavioral science. Frontiers in Psychology.

[B67-behavsci-16-01343] Snyder H. (2019). Literature review as a research methodology: An overview and guidelines. Journal of Business Research.

[B68-behavsci-16-01343] Sovacool B. K. (2014). What are we doing here? Analyzing fifteen years of energy scholarship and proposing a social science research agenda. Energy Research & Social Science.

[B69-behavsci-16-01343] Sovacool B. K., Kivimaa P., Hielscher S., Jenkins K. (2019). Further reflections on vulnerability and resistance in the United Kingdom’s smart meter transition. Energy Policy.

[B70-behavsci-16-01343] Stauch A., Gamma K. (2020). Cash vs. solar power: An experimental investigation of the remuneration-related design of community solar offerings. Energy Policy.

[B71-behavsci-16-01343] Steg L., Perlaviciute G., Van der Werff E. (2015). Understanding the human dimensions of a sustainable energy transition. Frontiers in Psychology.

[B72-behavsci-16-01343] Steg L., Perlaviciute G., Van der Werff E., Lurvink J. (2014). The significance of hedonic values for environmentally relevant attitudes, preferences, and actions. Environment and Behavior.

[B73-behavsci-16-01343] Stern P. C. (2000). New environmental theories: Toward a coherent theory of environmentally significant behavior. Journal of Social Issues.

[B74-behavsci-16-01343] Stern P. C., Dietz T., Abel T., Guagnano G. A., Kalof L. (1999). A value-belief-norm theory of support for social movements: The case of environmentalism. Human Ecology Review.

[B75-behavsci-16-01343] Streimikiene D., Baležentis T., Volkov A., Morkūnas M., Žičkienė A., Streimikis J. (2021). Barriers and drivers of renewable energy penetration in rural areas. Energies.

[B76-behavsci-16-01343] Sugihara C., Hardman S. (2022). Electrifying California fleets: Investigating light-duty vehicle purchase decisions. Transportation Research Interdisciplinary Perspectives.

[B77-behavsci-16-01343] Thunshirn P., Mlinaric I., Berg J. (2025). A qualitative analysis of consumer motivations and barriers towards active smart meter utilization. Energy Policy.

[B78-behavsci-16-01343] Tirotto F. A., Pahl S. (2023). Exploring the relationship between group-level autonomy and social acceptance of geothermal energy: The role of procedural justice climate, social identity violation, and collective self-determined motivation. Psicologia Sociale.

[B79-behavsci-16-01343] Tranfield D., Denyer D., Smart P. (2003). Towards a methodology for developing evidence-informed management knowledge by means of systematic review. British Journal of Management.

[B80-behavsci-16-01343] Tricco A. C., Lillie E., Zarin W., O’Brien K. K., Colquhoun H., Levac D., Moher D., Peters M. D. J., Horsley T., Weeks L., Hempel S., Akl E. A., Chang C., McGowan J., Stewart L., Hartling L., Aldcroft A., Wilson M. G., Garritty C., Straus S. E. (2018). PRISMA extension for scoping reviews (PRISMA-ScR): Checklist and explanation. Annals of Internal Medicine.

[B81-behavsci-16-01343] van der Werff E., Steg L., Keizer K. (2013). The value of environmental self-identity: The relationship between biospheric values, environmental self-identity and environmental preferences, intentions and behaviour. Journal of Environmental Psychology.

[B82-behavsci-16-01343] Vansteenkiste M., Ryan R. M., Soenens B. (2020). Basic psychological need theory: Advancements, critical themes, and future directions. Motivation and Emotion.

[B83-behavsci-16-01343] Venkatesh V., Morris M. G., Davis G. B., Davis F. D. (2003). User acceptance of information technology: Toward a unified view1. MIS Quarterly.

[B84-behavsci-16-01343] Vorobeva D., Scott I. J., Oliveira T., Neto M. (2026). Bridging individual and collective action: Advancing energy community participation through demand response tools. Energy Reports.

[B85-behavsci-16-01343] Wang S., Wang Y., Fan S., Shahbaz M., Guo X., Yu R. (2024). Does residential photovoltaic installation matter for coal-to-electricity transition? Evidence from self-determination theory perspective. Current Psychology.

[B86-behavsci-16-01343] Wang Y., Gu C., Xie D., Alhazmi M., Kim J., Wang X. (2025). Enhancing peer-to-peer energy trading in Integrated Energy Systems: Gamified engagement strategies and differentiable robust optimization. Energy Reports.

[B87-behavsci-16-01343] Webb D., Olaru D., Rastin C. (2025). Unlocking motivation for energy saving: A study of German electricity consumer segments. Energy Research & Social Science.

[B88-behavsci-16-01343] Whitmarsh L., O’Neill S. (2010). Green identity, green living? The role of pro-environmental self-identity in determining consistency across diverse pro-environmental behaviours. Journal of Environmental Psychology.

[B89-behavsci-16-01343] Wohlin C. (2014). Guidelines for snowballing in systematic literature studies and a replication in software engineering. Proceedings of the 18th international conference on evaluation and assessment in software engineering.

[B90-behavsci-16-01343] Wunderlich P., Kranz J., Totzek D., Veit D., Picot A. (2013). The impact of endogenous motivations on adoption of IT-enabled services: The case of transformative services in the energy sector. Journal of Service Research.

[B91-behavsci-16-01343] Wunderlich P., Veit D. J., Sarker S. (2019). Adoption of sustainable technologies: A mixed-methods study of German households. MIS Quarterly.

[B92-behavsci-16-01343] Wüstenhagen R., Wolsink M., Bürer M. J. (2007). Social acceptance of renewable energy innovation: An introduction to the concept. Energy Policy.

[B93-behavsci-16-01343] Zhang M., Eliassen F., Taherkordi A., Jacobsen H. A., Li Y., Zhang Y. (2025). Self-determination theory and deep reinforcement learning for personalized energy trading in smart grid. IEEE Transactions on Systems, Man, and Cybernetics: Systems.

[B94-behavsci-16-01343] Zhao A. P., Li S., Alhazmi M., Bao Z., Cheng X. (2025). Psychological insights for electric vehicles. International Journal of Electrical Power & Energy Systems.

[B95-behavsci-16-01343] Zhao H., Furuoka F., Rasiah R. (2024). The influence of psychological factors on consumer purchase intention for electric vehicles: Case study from China: Integrating the necessary condition analysis methodology from the perspective of self-determination theory. World Electric Vehicle Journal.

